# Isolation and characterization of IgG3 glycan-targeting antibodies with exceptional cross-reactivity for diverse viral families

**DOI:** 10.1371/journal.ppat.1012499

**Published:** 2024-09-18

**Authors:** Matthew J. Vukovich, Andrea R. Shiakolas, Jared Lindenberger, Robert A. Richardson, Lindsay E. Bass, Maggie Barr, Yanshun Liu, Eden P. Go, Chan Soo Park, Aaron J. May, Salam Sammour, Chipo Kambarami, Xiao Huang, Katarzyna Janowska, Robert J. Edwards, Katayoun Mansouri, Taylor N. Spence, Alexandra A. Abu-Shmais, Nelia P. Manamela, Simone I. Richardson, Sabina E. W. Leonard, Kathryn R. Gripenstraw, Ian Setliff, Kevin O. Saunders, Rachel H. Bonami, Ted M. Ross, Heather Desaire, Penny L. Moore, Robert Parks, Barton F. Haynes, Daniel J. Sheward, Priyamvada Acharya, Giuseppe A. Sautto, Ivelin S. Georgiev

**Affiliations:** 1 Vanderbilt Vaccine Center, Vanderbilt University Medical Center, Nashville, Tennessee, United States of America; 2 Department of Pathology, Microbiology and Immunology, Vanderbilt University Medical Center, Nashville, Tennessee, United States of America; 3 Duke Human Vaccine Institute, Durham, North Carolina, United States of America; 4 Florida Research and Innovation Center, Cleveland Clinic, Port Saint Lucie, Florida, United States of America; 5 Center for Vaccines and Immunology, University of Georgia, Athens, Georgia, United States of America; 6 Department of Chemistry, University of Kansas, Lawrence, Kansas, United States of America; 7 Department of Biochemistry, Duke University School of Medicine, Durham, North Carolina, United States of America; 8 MRC Antibody Immunity Research Unit, School of Pathology, University of the Witwatersrand, Johannesburg, South Africa; 9 National Institute for Communicable Diseases of the National Health Laboratory Service, Johannesburg, South Africa; 10 Program in Chemical and Physical Biology, Vanderbilt University Medical Center, Nashville, Tennessee, United States of America; 11 Division of Rheumatology and Immunology, Department of Medicine, Vanderbilt University Medical Center, Nashville, Tennessee, United States of America; 12 Vanderbilt Center for Immunobiology, Vanderbilt University Medical Center, Nashville, Tennessee, United States of America; 13 Vanderbilt Institute for Infection, Immunology and Inflammation, Vanderbilt University Medical Center, Nashville, Tennessee, United States of America; 14 Department of Infectious Diseases, University of Georgia, Athens, Georgia, United States of America; 15 Department of Medicine and Immunology, Duke University School of Medicine, Durham, North Carolina, United States of America; 16 Department of Microbiology, Tumor and Cell Biology, Karolinska Institutet, Stockholm, Sweden; 17 Department of Surgery, Duke University School of Medicine, Durham, North Carolina, United States of America; 18 Department of Computer Science, Vanderbilt University, Nashville, Tennessee, United States of America; 19 Center for Structural Biology, Vanderbilt University, Nashville, Tennessee, United States of America; 20 Program in Computational Microbiology and Immunology, Vanderbilt University Medical Center, Nashville, Tennessee, United States of America; University of Wisconsin, UNITED STATES OF AMERICA

## Abstract

Broadly reactive antibodies that target sequence-diverse antigens are of interest for vaccine design and monoclonal antibody therapeutic development because they can protect against multiple strains of a virus and provide a barrier to evolution of escape mutants. Using LIBRA-seq (linking B cell receptor to antigen specificity through sequencing) data for the B cell repertoire of an individual chronically infected with human immunodeficiency virus type 1 (HIV-1), we identified a lineage of IgG3 antibodies predicted to bind to HIV-1 Envelope (Env) and influenza A Hemagglutinin (HA). Two lineage members, antibodies 2526 and 546, were confirmed to bind to a large panel of diverse antigens, including several strains of HIV-1 Env, influenza HA, coronavirus (CoV) spike, hepatitis C virus (HCV) E protein, Nipah virus (NiV) F protein, and Langya virus (LayV) F protein. We found that both antibodies bind to complex glycans on the antigenic surfaces. Antibody 2526 targets the stem region of influenza HA and the N-terminal domain (NTD) region of SARS-CoV-2 spike. A crystal structure of 2526 Fab bound to mannose revealed the presence of a glycan-binding pocket on the light chain. Antibody 2526 cross-reacted with antigens from multiple pathogens and displayed no signs of autoreactivity. These features distinguish antibody 2526 from previously described glycan-reactive antibodies. Further study of this antibody class may aid in the selection and engineering of broadly reactive antibody therapeutics and can inform the development of effective vaccines with exceptional breadth of pathogen coverage.

## Author summary

Antibodies typically recognize antigen with high specificity, thus targeting a single pathogen. We used LIBRA-seq to identify a class of glycan-targeting human IgG3 antibodies with antigen specificities that span multiple viral families including influenza HA, HIV-1 Env, and SARS-CoV-2 spike, while simultaneously displaying no measurable autoreactivity to human proteins. One of these antibodies, mAb 2526, contains a glycan-binding pocket on the light chain, recognizes complex glycans on the antigenic surface and cross-reacts with antigens from multiple pathogens.

## Introduction

Identification of broadly reactive antibodies has been instrumental in the design of vaccines and therapeutics against highly diverse pathogens. In the field of HIV-1 vaccine design, the discovery of broadly neutralizing antibodies (bNAbs) and identification of their epitopes have allowed for reverse vaccinology approaches to expand vaccine-elicited neutralization breadth [1–3]. The discovery and characterization of new classes of broadly reactive antibodies can provide new pathways for the development of broadly protective antibody-based vaccines and therapeutics [4–7].

Broadly-reactive antibodies that target diverse strains within a virus species have been isolated from numerous individuals [8–12]. These include broadly neutralizing antibodies such as PGT145, 1707, and 71281–33 that target multiple strains of HIV-1 Env, influenza HA, and SARS-CoV-2 spike respectively [8–10]. Bi-and tri-specific antibodies have been engineered to further expand breadth, sometimes reaching pan-neutralization within a viral species [13,14]. Antibodies of this nature typically target protein epitopes while also making key interactions with glycans on the antigenic surface [15–18]. Other multi-specific antibodies exhibit much broader cross-reactivity, recognizing antigens from different virus species including HIV-1 Env, influenza HA, and coronavirus spike [19,20]. Multi-specific antibodies of this nature are much rarer and target commonalities that exist between unrelated pathogens, thus revealing exploitable vulnerabilities of these pathogens. Antibodies of this class, typified by the HIV-1 bNAb 2G12, tend to recognize primarily high mannose glycans on the antigenic surface, although antibodies that cross-react with HIV-1 and HCV in a glycan-independent manner have been identified [19–21].

Viruses often employ host glycosylation machinery to post-translationally modify their antigens to evade the host immune response [22–24]. Host tolerance mechanisms present a significant roadblock to the elicitation of glycan-reactive antibodies; thus, glycosylation of antigens acts to shield the protein surface of an antigen from antibody recognition. Even so, several glycan-reactive antibodies have recently been discovered including Fab-dimerized glycan reactive antibodies (FDGs), which take on a unique “I” shape and target high-mannose glycans, much like 2G12 [19]. Recently our lab discovered and characterized a glycan-targeting IgG3 antibody isolated from a donor chronically infected with both HIV-1 and HCV, antibody 688 [20,25]. Since the addition of glycans to antigens is conserved across many viral species, targeting viral glycans is a valuable strategy for developing therapeutics that can be used against future pathogens. Glycans that are targeted by such antibodies, however, are also found on host proteins and pose the risk of inducing an autoreactive response. The complexity of this problem is illustrated by the high rates of autoreactivity seen in several FDG antibodies [19].

Here we report the discovery and characterization of an IgG3 class of glycan-reactive antibodies that possess distinct antigen reactivity profiles compared to previously described glycan-targeting antibodies. We mined a published LIBRA-seq dataset whereby the B cell repertoire of an individual who had been chronically infected with HIV-1 was screened for reactivity against a diverse panel of HIV-1 Env and influenza HA antigens [9]. We specifically searched for and identified B cells with positive LIBRA-seq scores (LSS) for at least one strain of HIV-1 Env and one strain of influenza HA. Notably, among the identified B cells with high LSS for both HIV-1 Env and influenza HA antigens, there were three that were clonally related and belonged to the same antibody lineage. Our results demonstrate that antibodies from this lineage recognize complex glycans on the surface of diverse antigens, including HIV-1 Env, influenza HA, coronavirus spike, HCV E, NiV F, and LayV F, while simultaneously showing no signs of autoreactivity. A crystal structure of 2526 IgG1 Fab bound to mannose revealed a glycan-binding pocket on the light chain, presenting a previously unappreciated mode of antibody-antigen recognition. Taken together, the discovery and study of this class of glycan-reactive antibodies offers new insights into unique modes of antigen recognition that may be amenable to optimization of vaccine design and therapeutic antibody development to either broaden or narrow the targeted antibody response.

## Results

### Discovery and characterization of cross-reactive antibodies from a chronically infected HIV-1 donor

**Fig 1:**
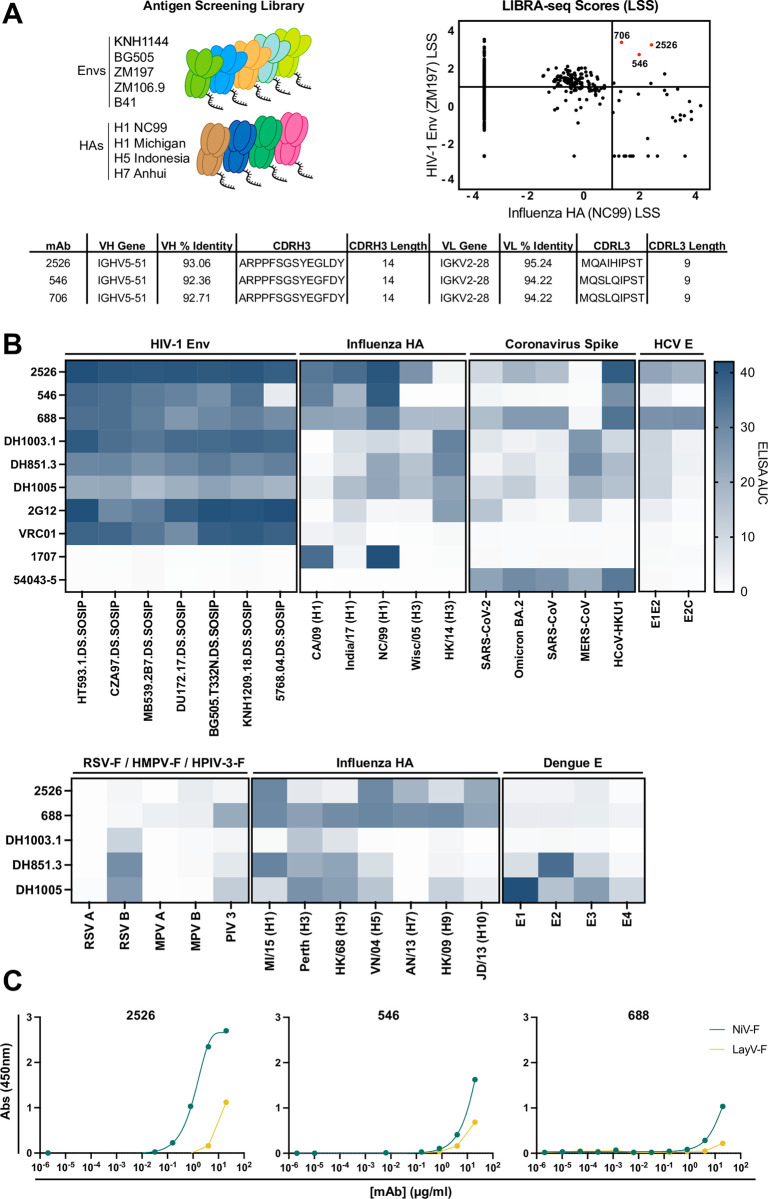
Discovery and validation of broadly-reactive mAbs 2526 and 546. (A) A diverse panel of HIV-1 Envs and influenza HAs were used to screen B cell receptor specificity of a donor chronically infected with HIV-1. B cell LIBRA-seq scores (LSS) for HIV-1 Env ZM197 and influenza HA NC99 identified potential broadly-reactive antibodies; red dots indicate antibodies 2526, 546, and 706. Sequence analysis revealed that all three antibodies came from the same lineage. VH and VL gene usage, CDR length, percent amino acid identity to germline, and CDR sequence for antibodies are shown. (B) 2526 and 546 cross-reactivity was validated against a diverse panel of antigens by ELISA and reactivity was compared to previously described cross-reactive mAbs. Representative ELISA area under the curve (AUC) from a set of three repeats are displayed. (C) 2526, 546, and mAb 688 bind to Nipah virus F (NiV-F) protein, and to a lesser extent Langya virus F (LayV-F) protein. Representative ELISA curves are shown.

We previously reported on the development of LIBRA-seq technology to screen for B cell receptor specificity against diverse antigen panels in a high throughput manner. This initial LIBRA-seq application involved probing the B cell repertoire of donor N90, an individual with chronic HIV-1 infection, against a panel of antigens including 5 HIV-1 Env antigens (KNH1144, BG505, ZM197, ZM106.9, and B41) and 4 influenza HA antigens (H1 NC99, H1 Michigan, H5 Indonesia, and H7 Anhui) [9,25]. From this LIBRA-seq experiment, we identified a set of three clonally related B cells that had positive LIBRA-seq scores for both influenza HA (H1 NC99) and HIV-1 Env (ZM197) antigens (Fig 1A). The clonality of these three B cells suggested that their predicted cross-reactivity was unlikely to be false positive, justifying their further study. We prioritized two antibodies (2526 and 546) with the most promising LIBRA-seq scores from this set of three for further experiments. We expressed 2526 and 546 in their native IgG3 isotype and validated their binding to a diverse panel of antigens in ELISA (Fig 1B). We further validated the breadth of 2526 against a panel of influenza HAs from H5N1 strains isolated from infected avian species and infected human species, along with H2N2 strains (S1 Fig). Both 2526 and 546 displayed antigen specificity profiles that are distinct from previously reported FDG cross-reactive antibodies (DH1003.1, DH851.3, DH1005, and 2G12). We then measured binding to Nipah virus and Langya virus fusion proteins (Fig 1C). Both 2526 and 546 showed reactivity to NiV-F, and to a lesser extent, LayV-F [26].

### 2526 and 546 bind to complex glycans on the surface of antigens

**Fig 2:**
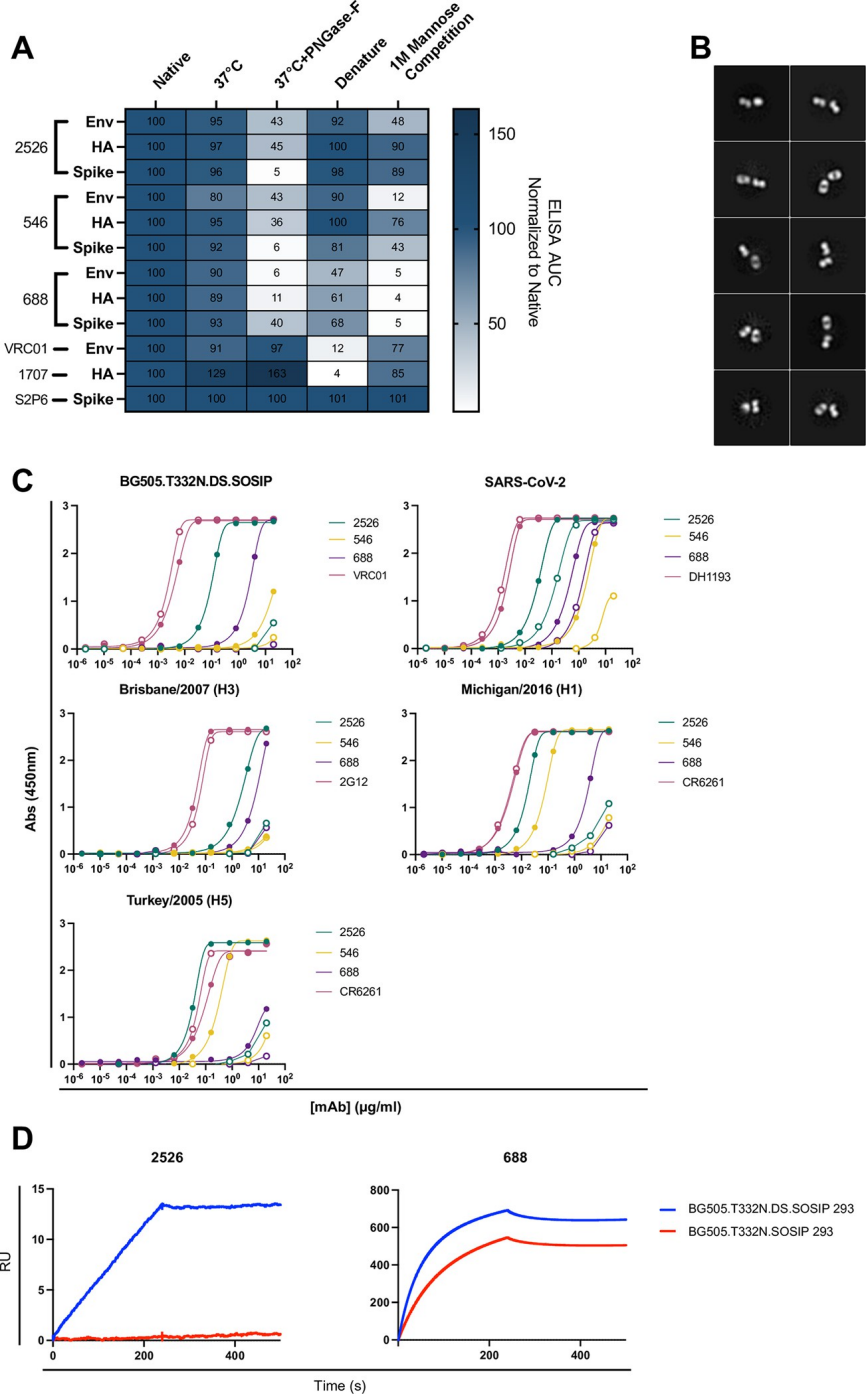
2526 and 546 bind to complex glycans on their target antigens. (A) 2526 and 546 were shown to be glycan reactive by enzymatic treatment of antigens with PNGase-F to remove N-linked glycans or competition with 1M mannose in an ELISA format. ELISA AUC normalized to native antigen is shown. Representative ELISA area under the curve (AUC) from a set of three repeats are displayed. (B) Negative stain electron microscopy confirmed that 2526 takes on the canonical “Y” shape of antibodies instead of the “I” shape of FDG antibodies. (C) 2526, 546, and 688 show a decrease in binding to antigen made in GnT1- cells, suggesting that they preferentially target complex glycans. Closed circles indicate the specified antibody was tested against antigen made in 293F cells while open circles indicate the antibody was tested against antigen made in GnT1- cells. (D) 2526 prefers to bind to BG505.DS.SOSIP compared to BG505.SOSIP when tested with SPR. Representative SPR curves from a set of two repeats are shown.

Previously reported cross-reactive antibodies have been found to react with glycans on the surface of their target antigens [19,20]. To test if this antibody family targets glycans, we treated various viral glycoprotein antigens with PNGase-F to remove N-linked glycans and measured binding in ELISA compared to untreated antigens (Fig 2A). Binding of 2526 and 546 to HIV-1 Env, influenza HA, and CoV spike was significantly decreased following treatment of antigen with PNGase-F. A potential confounding factor in this assay is that glycans can serve as important mediators of protein structure, and their removal can distort an antigen’s conformation and alter antibody binding as a result. To address this possibility, we intentionally disrupted the conformation of antigen by mixing native antigen with a denaturing and reducing buffer and boiling the mixture at 95°C for 10 minutes prior to the ELISA. Under these denaturing conditions, binding of antibodies 2526 and 546 to HIV-1 Env, influenza HA, and SARS-CoV-2 spike was mostly unaffected with negligible decreases in some cases. In contrast, binding of antibody 688 to all three antigens was slightly decreased, potentially indicating some degree of reliance on antigen conformation. VRC01 and 1707 binding was completely ablated upon denaturing HIV-1 Env and influenza HA respectively. Taken together, this data suggests that the decrease in binding of 2526 and 546 following PNGase-F treatment of antigen is due to removal of N-linked glycans on the antigen and not a disruption in protein structure.

Several previously described glycan-reactive antibodies are known as FDG in that their Fab regions dimerize to give the antibodies a distinct “I” shape. We used negative-stain electron microscopy to visualize 2526 and found it to be in the canonical “Y” shape, excluding it from the FDG category (Fig 2B). N-linked glycans are heterogeneous in nature, some are moderately processed and consist primarily of mannose subunits (high mannose) whereas others are further processed into more hybrid or complex forms with additional sugars moieties added. To better understand the binding of 2526 and 546 to the glycans on their target antigens, we sought to identify which type of glycans were targeted, using either 293F or GnT1- cell lines to produce antigens. The GnT1- cell line lacks N-acetylglucosaminyltransferase activity and therefore lacks complex N-linked glycans. ELISA measurements of binding to antigens made in either cell type revealed that binding of 2526 and 546 was heavily dependent on the presence of complex glycans, suggesting that these antibodies interact with complex glycans on the antigen surface (Fig 2C). This pattern was observed for all antigens from three viral families (HIV-1 Env, influenza HA, and CoV spike) and was replicated using SPR as an additional assay for binding (S2 Fig). Antigens produced in GnT1- cells were validated to have a smaller size using mass photometry (S3 Fig). In addition to preferential targeting of complex glycans over high mannose-type, we found that 2526 and 688 showed increased binding to the DS.SOSIP version [27] of HIV-1 BG505 Env over the SOSIP version (Fig 2D). We compared the site-specific glycosylation profile of the BG505.T332N.DS.SOSIP with the BG505.T332N.SOSIP and found that they were largely identical with the exception of glycans 262 and 276. Glycan 262 was found to exist in a complex form at a higher proportion in the DS.SOSIP than what is measured in the SOSIP version. Conversely, glycan 276 was found to exist in a less processed glycoform in the DS.SOSIP version compared to the SOSIP Env (S4 Fig). Since 2526 showed broader reactivity than 546, we focused the remaining efforts on antibody 2526.

### 2526 does not display signs of autoreactivity

**Fig 3:**
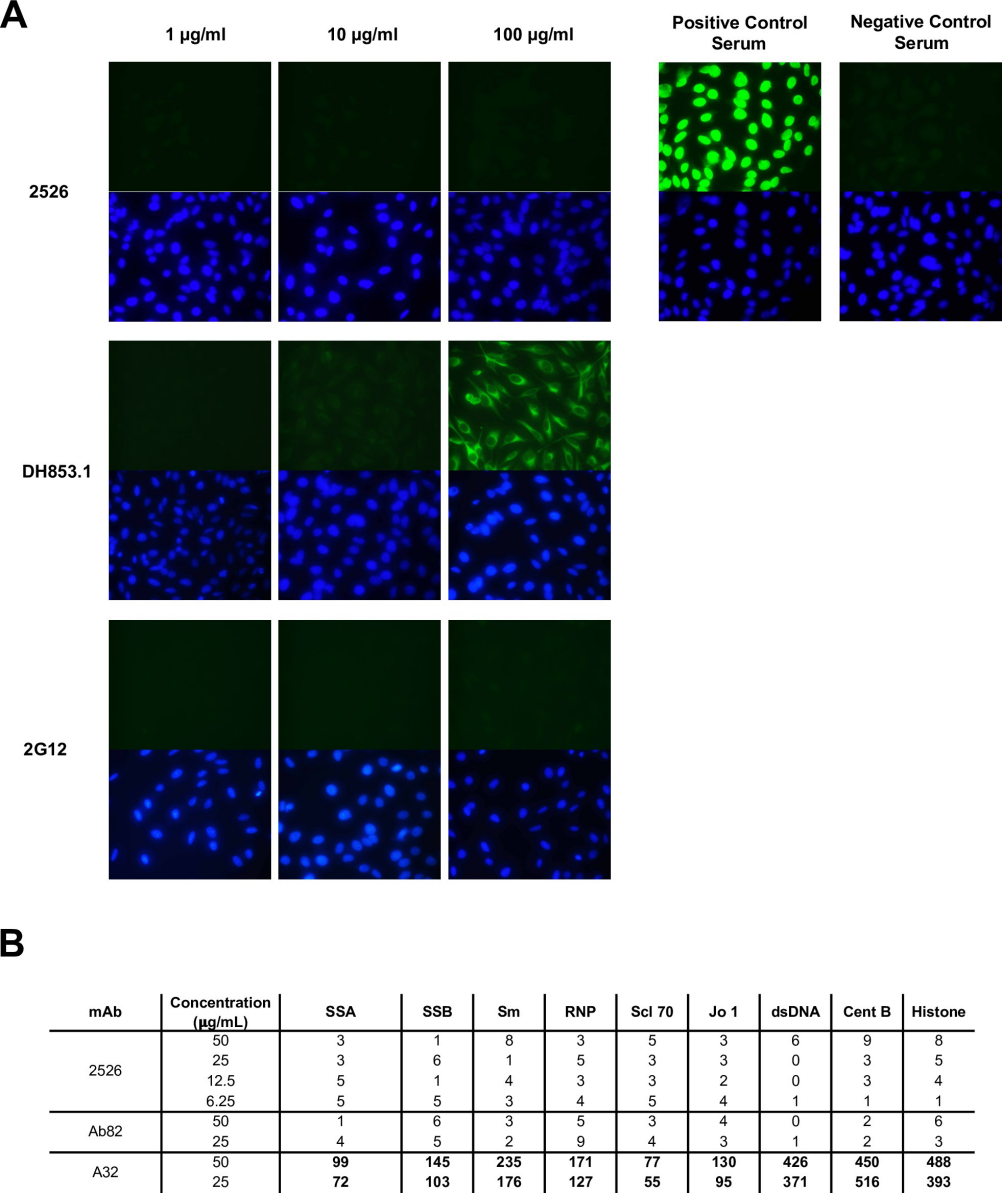
2526 does not display signs of autoreactivity. (A) HEp-2 cell slides were immunofluorescently stained with the indicated mAbs and goat anti-human Ig-FITC secondary (green) and DAPI (blue, to stain cell nuclei). Each mAb was tested at 100, 10, and 1 μg/mL. Control human serum samples that were positive or negative for anti-nuclear antibodies are shown (right). Images are 40X magnification. (B) Autoantigens were tested for reactivity by 2526, Ab82 (negative control antibody), and A32 (positive control antibody) using a commercially available AtheNA Multi-Lyte ANA kit. Values shown in bold were considered positive.

A common feature among broadly reactive antibodies is some degree of autoreactivity to self-proteins [28,29]. With glycan-reactive antibodies, this feature may be expected to be more pronounced in that both viral proteins and host proteins are glycosylated through identical mechanisms. Due to the broad antigen specificity of 2526, we tested for autoreactivity to HEp-2 cells as well as against a standard panel of nuclear proteins (Fig 3). While the FDG antibody DH853.1 showed staining of human proteins expressed by HEp-2 cells at the highest mAb concentration tested, 2526 showed no measurable autoreactivity, even when tested at 100 μg/ml (Fig 3A). Next, we assessed binding of 2526 to a standardized panel of nuclear proteins and found no signs of autoreactivity (Fig 3B). Although glycans on these nuclear antigens may be absent, some previously described glycan-reactive antibodies have been reported to react to a subset of these antigens [19]. Taken together, 2526 shows no signs of autoreactivity and exhibits a higher degree of specificity for viral antigens over human proteins, which contrasts that of DH853.1 in this study as well as other FDGs in previous work [19].

### 2526 contains a glycan-binding pocket in the light chain

**Fig 4:**
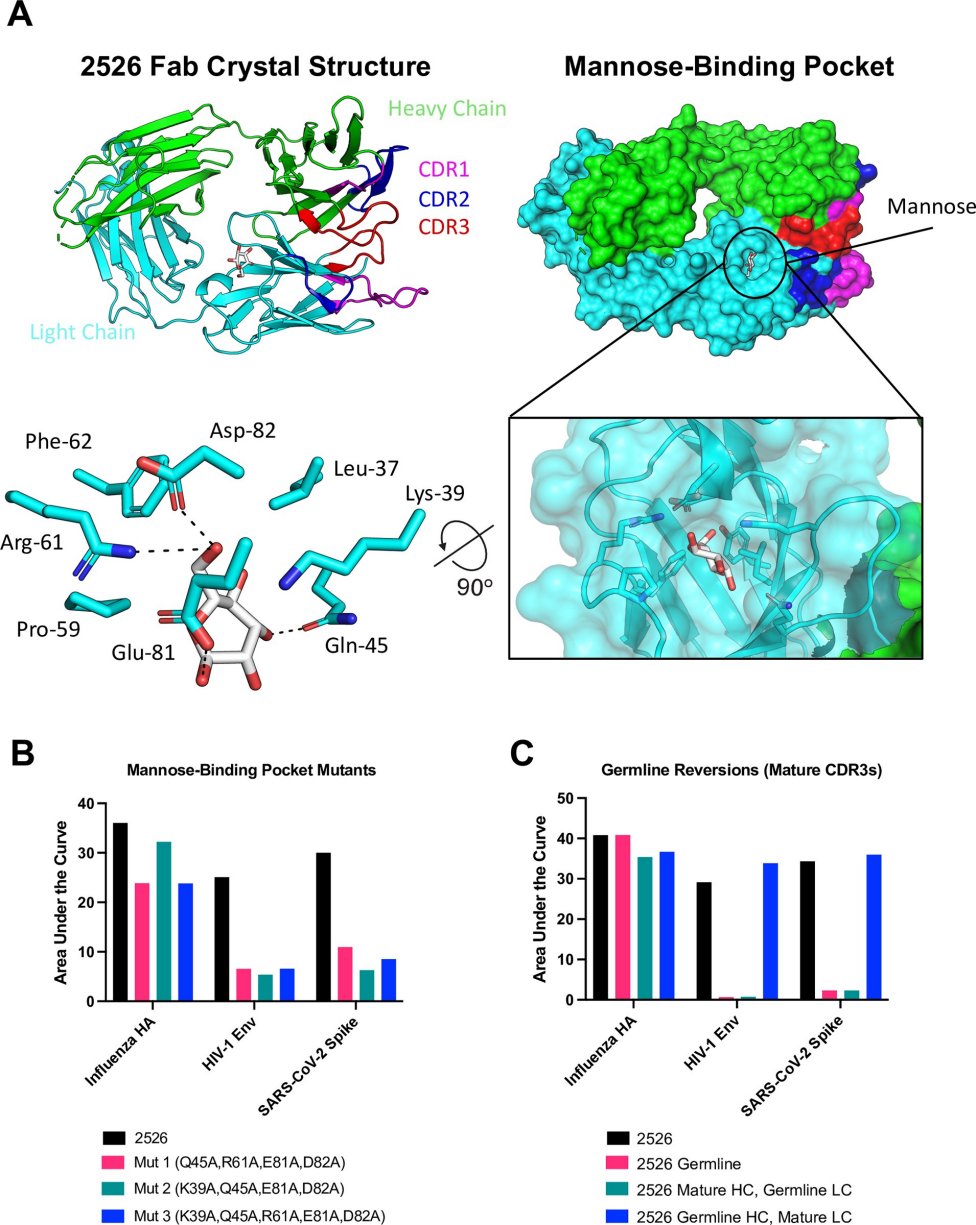
X-ray crystallography identified a functional glycan-binding pocket on the side of the light chain of 2526. (A) Crystal structure of 2526 IgG1 Fab complexed with D-Mannose is shown. The light chain is shown in cyan and the heavy chain is shown in green. Pink, blue, and red denote CDR loops. D-Mannose is shown in white and red, with its binding pocket in the crystal structure highlighted in surface representation (right) with key amino acid residues interacting with the mannose moiety shown as sticks (bottom). (B) Mutating residues in the binding pocket decreased binding of 2526 IgG3 to HIV-1 Env (KNH1209.18.DS.SOSIP), SARS-CoV-2 spike (Index strain), and influenza HA (Michigan/2016 H1). (C) Germline reversion of 2526 IgG3, whereby the amino acid sequences of the heavy and light chain were reverted to their inferred germline sequences whilst keeping the CDR3 regions mature, ablated binding to HIV-1 Env (KNH1209.18.DS.SOSIP) and SARS-CoV-2 spike (Index strain), but not to influenza HA (New Caledonia/1999 H1). Keeping the light chain mature while reverting the heavy chain to germline rescued binding. HC, heavy chain. LC, light chain. Representative ELISA area under the curve (AUC) from a set of three repeats are displayed.

We next sought to define the structure of the Fab domain of 2526 to better understand its mechanism of antigen binding. To identify the binding site and define interactions between sugars and 2526 at a high resolution, the fragment antigen-binding (Fab) of 2526 IgG1 was co-crystallized with mannose, and a 1.8 Å crystal structure was determined (Fig 4). Mannose was observed bound within a light chain pocket formed by residues within framework regions 2 and 3 including: Lys 39, Gln 45, Arg 61, Glu 81, and Asp 82 (Fig 4A). The mannose binding pocket was stabilized by interactions with the adjacent light chain complementarity determining region 2 (LCDR2) (S5 Fig). Mutant 2526 IgG3 antibodies, whereby combinations of the residues lining the mannose binding pocket identified in the crystal structure were mutated to alanine, displayed dramatically reduced binding to HIV-1 Env, SARS-CoV-2 spike, and influenza HA (Fig 4B). This data implicated the glycan-binding pocket of the light chain as the primary influence on binding to HIV-1 Env, SARS-CoV-2 spike, and influenza HA. We then produced germline-reverted versions of 2526 IgG3, whereby either one or both the heavy and light chain sequences were reverted to their inferred germline sequence, except for the CDR3 regions which were kept in the mature form (Fig 4C). Germline 2526 IgG3 retained binding to influenza HA but lost binding to both HIV-1 Env and SARS-CoV-2 spike, indicating that mutations obtained during affinity maturation are essential for binding to Env and spike. We note that the essential residues that make up the glycan-binding pocket are present in the germline sequences, thus suggesting that there are additional residues that are critical for HIV-1 Env and SARS-CoV-2 spike binding in addition to those that make up this glycan-binding pocket. Interestingly, when the mature light chain of 2526 was paired with the germline heavy chain, binding was retained for all three antigens. The reverse setup, where the mature heavy chain was paired with a germline light chain, showed a dramatic reduction/loss of binding for all three antigens (Fig 4C). Taken together, the light chain of 2526 appears to be most important for binding to HIV-1 Env and SARS-CoV-2 spike, and key residues for such binding include affinity matured residues along with germline-present residues that make interactions within the glycan-binding pocket. Since our data indicates that 2526 requires complex glycans on antigens for binding, it is likely that there are additional interactions between 2526 and its target antigens besides those identified in the structure of 2526 IgG1 Fab bound to mannose.

### 2526 isotype is important for antigen recognition

Previous studies have shown that the isotype of an antibody can dramatically alter binding [20,30–32]. We expressed and purified 2526 as an IgG1 isotype and compared its binding in ELISA to that of the IgG3 version (S6 Fig). Binding of all antigens tested showed a substantial decrease in binding of the IgG1 version of 2526 compared to the IgG3 version, suggesting that the IgG3 isotype of 2526 imparts features that are critical to its binding. The longer hinge region of IgG3 antibodies may play a role in this increase in binding, potentially allowing for more flexibility in the antigen-binding arms giving rise to a more avid interaction with antigen.

### 2526 recognizes distinct antigenic epitopes and its binding is modulated by removal of glycans

**Fig 5:**
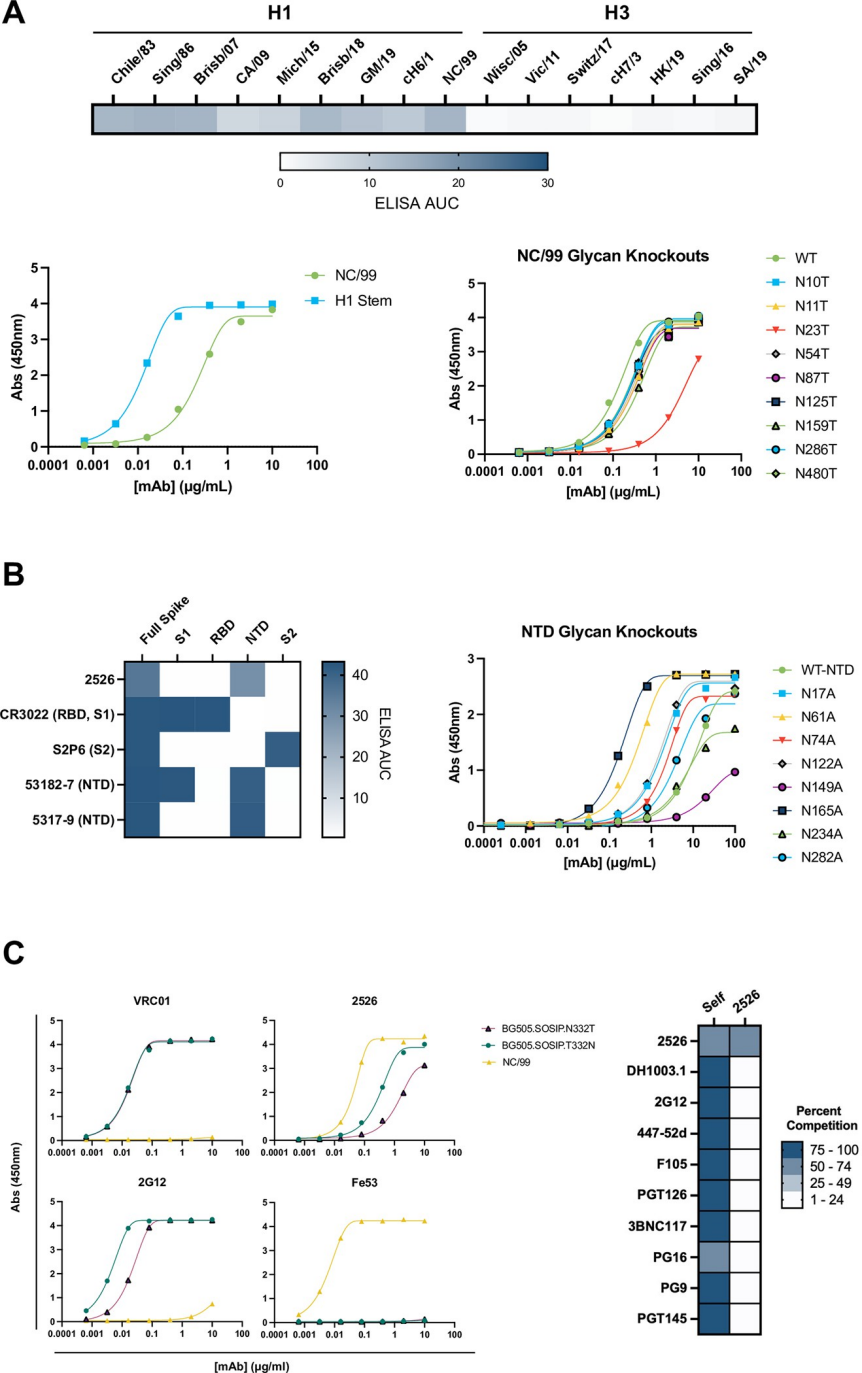
2526 Epitope Mapping for influenza HA, SARS-CoV-2 spike, and HIV-1 Env. (A) 2526 was tested against an expanded panel of influenza A HA strains in ELISA and was found to preferentially bind to H1 HAs compared to H3 HAs. Binding of 2526 to an H1 stem-only stabilized construct (H1 Stem) suggested that 2526 targets the stem region. Removal of the stem glycan N23 dramatically decreased binding. WT, wild type HA. (B) 2526 was tested against full SARS-CoV-2 spike as well as subunit constructs in an ELISA format, binding strongly to the full spike as well as the N-terminal domain (NTD) construct. RBD, receptor binding domain. NTD subunit constructs with individual glycans knocked out revealed a decrease in binding of 2526 to NTD when glycan 149 was removed. (C) 2526 was tested for binding to BG505.SOSIP with and without the presence of a glycan at position 332 (left). In a competition ELISA for 2526 against a panel of HIV-1 antibodies, 2526 displayed measurable competition against itself, but not competition against a panel of known HIV-1 antibodies (right).

We next sought to identify the epitopes targeted by 2526 on influenza HA, SARS-CoV-2 spike, and HIV-1 Env. We first tested 2526 for binding to an expanded panel of HA from influenza strains in ELISA, with a focus on H1 and H3 subtypes (Fig 5A). We found that 2526 binds stronger to HA from H1 subtypes compared to H3. Next, we tested binding of 2526 to an H1 stem-only construct and observed strong binding compared to the full H1 NC99 trimer (Fig 5A). We then designed and expressed a series of point mutants whereby individual glycans on the H1 NC/99 trimer were removed by mutating the asparagine residue in a potential N-linked glycosylation sequon to alanine. 2526 showed a dramatic reduction in binding when the sequon for glycan N23 was mutated, whereas mutating the sequon of all other glycans individually had no discernable effect compared to wild-type binding (Fig 5A). A previously established HA amino acid residue numbering scheme was used to assign position [33]. Aligned with the 2526 preference for H1 vs. H3 HAs, glycan N23 is strongly conserved across H1 subtypes of influenza but is absent from H3 subtypes. Previous work used tandem mass spectrometry to identify the site-specific glycosylation profile of the NC/99 HA and demonstrated that glycans at position 23 predominately exist in complex processed forms [34]. From these experiments, we concluded that 2526 likely makes contacts with the N23 glycan present on the stem region of H1 HA.

Next, we sought to define the epitope targeted on SARS-CoV-2 spike (Fig 5B). Testing of various spike subunits revealed that 2526 binds equally well to the NTD as compared to the full-length SARS-CoV-2 trimer, whereas no binding was observed for the other subunits. We note that although the NTD is present in the S1 subunit, we did not see binding to S1 by 2526 or an NTD-targeting control antibody, 5317–9. This phenotype has been previously reported for the 5317–9 antibody [35]. Comparatively, a different control antibody for NTD, 53182–7, bound to both the S1 subunit as well as the NTD subunit. While it is possible that there is differential glycosylation between the NTD region on the S1 subunit and the NTD subunit, antibody 5317–9 has not been investigated for glycan reactivity. It may be the case that the epitopes targeted by 2526 and 5317–9 are exposed on the NTD subunit but occluded on the S1 subunit. Individual glycan knockouts in NTD subunit constructs showed substantial changes in binding, with some glycan removals greatly enhancing binding (N165 and N61), whereas removal of N149 substantially reduced binding. The identity of the glycan that occupies position 149 on the SARS-CoV-2 spike has been reported to exist predominately in the complex form [23]. These results indicate that 2526 likely makes contacts with the N149 glycan in the NTD region of SARS-CoV-2 spike.

We then measured 2526 binding to the HIV-1 gp140 Env C.1086, as well as subunit constructs of C.1086, including the gp120 monomer and a V1V2 construct (S7A Fig). Strong binding was observed against all three constructs, suggesting that 2526 can bind to the V1V2 region of HIV-1 Env. Interestingly, however, knocking out glycans in the V1V2 region in the context of soluble trimer, both individually and collectively, had no effect on binding (S7B Fig). To address this, we expanded our mapping of the HIV-1 Env epitope to include the CD4 binding site bait (RSC3), a gp120 construct that lacks the V1, V2, and V3 regions (CH505 Con ΔV123), as well as a gp41 construct made in E.coli and thus lacking glycosylation. We observed binding to the RSC3 antigen as well as CH505 Con ΔV123 constructs but not the E.coli expressed gp41 construct (S7A Fig). Notably, binding to RSC3 and CH505 Con ΔV123 was lost upon removal of glycans with PNGase-F. Together, these data suggested that 2526 likely does not target the V1V2 region in the context of the soluble trimer. We then assessed whether removal of the glycan present at position 332 of the soluble trimer modulates binding. We found that 2526 binds notably worse to the BG505 SOSIP that lacks a glycan at position 332, similar to what is observed for antibody 2G12 (Fig 5C). Interestingly, 2526 was not found to compete with a panel of published HIV-1 antibodies, including 2G12 (Fig 5C). Overall, these results indicate that while the epitope of 2526 may be modulated by removal of the 332 glycan, the 2526 epitope likely does not significantly overlap with the 2G12 epitope which includes the 332 glycan.

### In vitro and in vivo functional characterization of 2526

**Fig 6:**
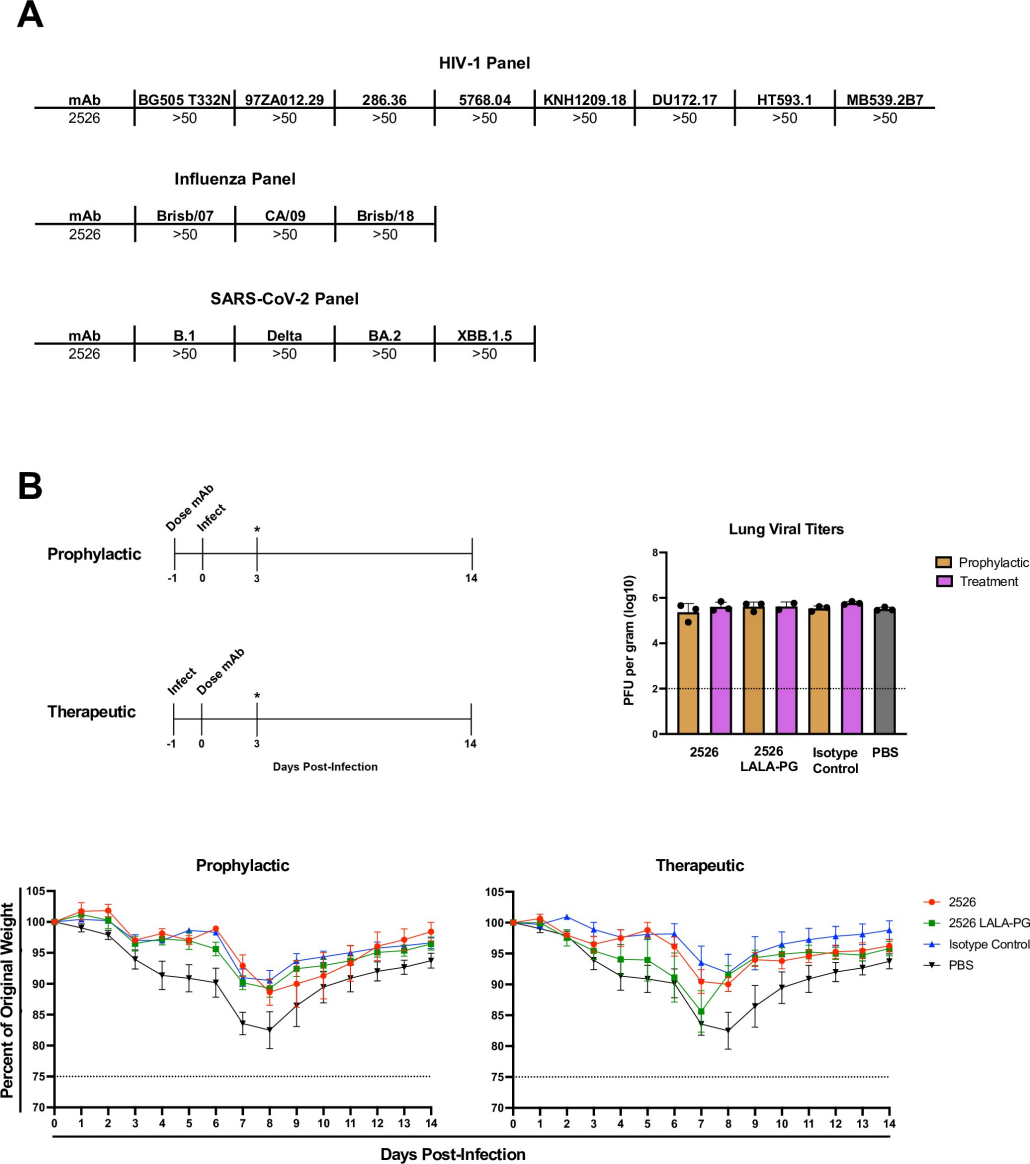
In-vitro and in-vivo functional characterization of 2526. (A) 2526 was found to be non-neutralizing against a panel of eight HIV-1 pseudovirus strains, three influenza strains, and four SARS-CoV-2 strains. (B) 2526 was tested for potential as a prophylactic and therapeutic agent in a mouse model of influenza challenge. A schematic of both study arms is shown with the H1N1 A/Brisbane/2018 influenza strain being administered prior to antibody treatment in the therapeutic arm and vice versa for the prophylactic arm. Lung viral titers were quantified 3 days post-infection (indicated by asterisk), with no significant differences between groups that received 2526 compared to negative control groups that received PBS or the isotype control. Comparisons of weight loss between groups showed that while mice receiving either 2526 or 2526 LALA-PG (Fc effector functions knocked out) did display less weight loss compared to mice receiving PBS, they did not perform better than mice that received a negative isotype control antibody that displays no reactivity to influenza antigens.

We measured the neutralization capability of 2526 against several strains of HIV-1, influenza, and SARS-CoV-2 virus (Fig 6A). In all cases we found 2526 to be non-neutralizing. Although 2526 did not neutralize influenza, we found that 2526 can recognize HAs purified from infectious influenza virions following split inactivation in a western blot format (S8 Fig). Recent work described non-neutralizing antibodies that confer in vivo protection in animal models of virus challenge [36]. We evaluated whether 2526 is protective in a mouse model of influenza challenge under both therapeutic and prophylactic conditions (Fig 6B). Under the prophylactic condition, mice were first dosed with either 2526, 2526 LALAPG (2526 with mutations that ablate Fc effector functions), an isotype negative control antibody, or PBS, and then subsequently challenged one day later with the H1N1 A/Brisbane/2018 influenza strain. For the therapeutic arm, mice were first challenged with A/Brisbane/2018 influenza and then subsequently treated (1 day post-challenge) with the same antibody groups listed above. Weight loss was monitored for 14 days post-infection and lung titers were assessed following day 3. Under both treatment arms, we did not observe a significant difference in either weight loss or lung viral titers between groups that received 2526 or 2526 LALAPG and the negative isotype control (Fig 6B).

## Discussion

Recent efforts have described several glycan-reactive antibodies that possess unusually broad specificity that can span multiple viral families [19,20]. We isolated and characterized a class of broad glycan-reactive antibodies with features that distinguish them from previously described cross-reactive antibodies. Antibody 2526 has a distinct antigen binding profile that includes several strains of HIV-1 Env, influenza HA, and CoV spike, as well as HCV E, NiV-F, and LayV-F. Distinct from the growing class of FDG antibodies that target high-mannose glycans, 2526 exists as a “Y” shaped IgG3 antibody that targets complex glycans. Characteristics of the IgG3 isotype impart unique features onto 2526 that are critical for its cross-reactivity. We found that 2526 binds significantly worse when expressed in the IgG1 isotype, possibly suggesting a role of the increased flexibility of IgG3 arms to allow for a higher avidity bivalent interaction compared to Fab alone or IgG1. Recent work has implicated IgG3 antibodies as having higher neutralization potential as well as potent complement activation, due to their ability to form high density clusters of IgG3-Fab due to the flexible hinge region that can display hexameric Fc platforms for docking of receptors and the complement C1 complex [37].

The structure of 2526 IgG1 Fab bound to mannose offers insight into a unique binding mechanism of glycan-reactive antibodies. We validated a glycan-binding pocket formed by residues contributed by the light chain framework regions 2 and 3, implicating key residues within the pocket to be necessary for binding to HIV-1 Env and SARS-CoV-2 spike. Since our data suggests that 2526 requires processing of antigenic glycans into more complex forms, this binding pocket likely comprises one of several interactions between the antibody and its target glycans, with critical contacts being made with the non-mannose subunits of glycans. It is also possible that the two Fab arms of the IgG3 are interacting with terminal mannose moiety of multiple glycan chains, with the IgG3 hinge conferring the ability to engage spatially separated glycans. The distance of the residues within the binding pocket from the CDRs on the heavy and light chain potentially offer opportunities to engineer the 2526 antibody to have desired binding properties without interfering with the glycan-binding pocket. We note that there is a possibility of allosteric modulation of the structure of the 2526 Fab as a result of binding to free mannose. This could have implications in identifying which residues are modifiable in steps to engineer the 2526 antibody to have desirable binding properties. We observed that modifying the 2526 antibody, whereby we reverted the heavy chain to an inferred germline form (with a mature HCDR3) while keeping the entire light chain mature, had negligible effects on binding to influenza HA, HIV-1 Env, and SARS-CoV-2 spike. This potentially offers more opportunities to engineer the 2526 antibody in that it may be possible to make changes to the 2526 heavy chain while retaining binding to desired antigens. We speculate that such changes to the heavy chain may make it possible to impart functions to the antibody that can eventually give it therapeutic and prophylactic use. We note that the ability of 2526 and 546 to bind to target antigens was decreased under the PNGase-F condition, but not completely lost. A possible explanation for this is that glycan removal is not complete under the conditions used in this study. Conversely, antigens made in GnT1- cells are more likely to uniformly lack complex glycan processing, resulting in a larger decrease in binding. While we observed a decrease in 2526 binding to SARS-CoV-2 spike made in GnT1- cells relative to 293F cells, binding to the spike produced in GnT1- cells was still measurable. This decrease in binding was more significant in the case of 2526 binding to HIV-1 Env or influenza HA, which showed a near complete loss in binding to antigens made in GnT1- cells. This could suggest that 2526 recognizes distinct glycan constellations on each antigen.

We found that 2526 likely targets glycans 23 and 149 on the influenza stem and SARS-CoV-2 spike, respectively, both of which have been found to exist in a complex-type glycoform [23,34]. We demonstrate that the epitope of 2526 is modulated by removal of glycan 332 on HIV-1 Env and likely does not overlap with the epitope of 2G12 which targets glycan 332. Paradoxically, we found that 2526 can bind to the V1V2 subunit construct whereas removal of glycans within the V1V2 region in the context of the trimer had no effect on binding. In this study we found that 2526 can bind to numerous different viral antigens tested, even though these different antigens have different arrays of glycans. With this in mind, it is not surprising that 2526 can bind to glycans on the V1V2 construct and does not necessarily imply that 2526 targets this region in the context of the trimeric antigen. Rather this may be an indication that testing binding to subunit constructs can introduce confounding results that require confirmation with glycan knockouts in the context of conformationally-relevant antigens.

Although 2526 exhibits promiscuous binding to antigens that span multiple viral families, we did not observe any measurable autoreactivity to self-proteins when tested against HEp-2 cells or a panel of nuclear proteins. This is in contrast with previously characterized glycan-reactive antibodies, including DH853.1, which showed measurable levels of reactivity against HEp-2 cells. We note that while 2526 did not display any measurable signs of autoreactivity in the assays used in this study, we cannot exclude the possibility that 2526 will react with host proteins that are absent from this assay or too low in amounts to detect. This characteristic of broad specificity towards viral antigens whilst restricting binding to self-proteins is highly sought after in therapeutics.

2526 was found to be non-neutralizing to HIV-1 Env pseudoviruses tested in this study. One possible explanation is that the epitope targeted by 2526 on the Env is not accessible until after Env engages with the CD4 receptor. Several previously studies antibodies have been found to have no detectable neutralization in assays that utilize standard TZM-bl cells, yet show detectable neutralization when TZM-bl/FcλRI cells are used [38]. These studies suggest that using TZM-bl/FcλRI cells positions antibodies at the cell surface and gives them a kinetic advantage, especially when such antibodies need to access epitopes that are more readily exposed after virus attachment. Another potential explanation is that the glycosylation of recombinant Env proteins used in this study differ from the glycosylation of Env on HIV-1 pseudoviruses. Several reports have demonstrated that while the site-specific glycosylation of recombinant SOSIP trimers largely mimics the glycosylation of trimers on virions, differential processing occurs and results in a higher proportion of complex-type glycans on virion-derived trimers compared to soluble trimers [39,40]. To this end, potential bias may have been introduced in the method of purification for Env trimers used in this study. The use of Galanthus Nivalis Lectin (GNL) can potentially select for Env trimers that have a higher proportion of high mannose-type glycans due to preferential binding to this glycoform. Cell-type used to express recombinant HIV-1 trimers has also been demonstrated to modulate the glycosylation profile of trimers [41]. An interesting observation that may further support a difference in glycosylation between recombinant and virion trimers is the fact that, in SPR measurements, 2526 binds stronger to BG505 SOSIPs that have the DS mutation set compared to BG505 SOSIPs that lack the DS mutation set (Fig 2D). The DS mutation set involves mutating Env residues 201 and 433 to cysteines, resulting in the introduction of a disulfide bond that prevents CD4 triggering/opening of the trimer [42]. One speculation to explain this phenomenon is that decreased flexibility of the DS-trimer can impact glycosylation and glycan processing, thus altering the binding of 2526. Although we found that the glycosylation profiles of the DS.SOSIP and SOSIP versions of the BG505 Env were largely conserved, there were notable differences between the levels of glycan processing at positions 262 and 276 (S4 Fig). Envs on pseudovirus do not have the DS mutation set, thus whatever differences exist between the DS and non-DS Envs that dictate binding and non-binding by 2526 may exist on the Envs on the pseudovirus versus the soluble DS Envs. On a similar note, we did not observe neutralization against several strains of influenza or SARS-CoV-2, and did not see protection against influenza in a mouse model of influenza challenge. These findings suggest that in its current state, 2526 would not be a feasible candidate for therapeutic or vaccine development. However, the observation that germline reversion of the heavy chain has no effect on binding to HIV-1 Env, influenza HA, and SARS-CoV-2 suggests that it may be possible to modulate regions of the heavy chain to increase the therapeutic potential of 2526. We highlight that 2526 was isolated from a sample collected prior to the beginning of the COVID-19 pandemic. Thus antibody 2526 reacts against the protein of a virus that was not circulating in the human population at the time of sample collection. This emphasizes the power of LIBRA-seq in identifying antibodies reactive against future circulating viruses, including pandemic viruses. Additionally, the unique features of antigen-recognition that 2526 possesses may be found in future glycan-reactive antibodies that have more favorable therapeutic profiles.

## Materials and methods

### Ethics statement

Mice were handled in accordance with protocols approved by the Cleveland Clinic Institutional Animal Care and Use Committee and were cared under U.S. Department of Agriculture guidelines for laboratory animals.

### LIBRA-seq data mining

The LIBRA-seq dataset generated in [9] was mined to identify antibodies that cross-react with influenza HA and HIV-1 Env. B cells with a LIBRA-seq score of at least one for a given antigen were considered positive.

### Antibody expression and purification

For each antibody, variable genes were inserted into plasmids encoding the constant region for the heavy chain and light chain and synthesized from Twist Biosciences. Antibodies were expressed in Expi293F mammalian cells (ThermoFisher) by transiently co-transfecting heavy and light chain plasmids in FreeStyle F17 expression media (ThermoFisher) supplemented to a final concentration of 0.1% Pluronic Acid F-68 and 20% 4 mM L-glutamine using Expifectamine transfection reagent (ThermoFisher Scientific) and cultured for five days at 8% CO_2_ saturation and 37°C with shaking. After five days, cultures were centrifuged at 4000 g for 20 minutes and filtered with Nalgene Rapid Flow Disposable Filter Units with PES membrane (0.22 μm). For IgG1 antibodies, the filtered supernatant was run over a column containing Protein A agarose resin that had been equilibrated with PBS. For IgG3 antibodies, the filtered supernatant was ran over a column containing Protein G agarose resin that had been equilibrated with PBS. Supernatant flow-through was saved and ran through the column again multiple times. The column was washed with PBS with two column volumes, after which antibodies were elute with 100 mM Glycine HCl at a pH of 2.7 directly into a 1:10 volume of 1 M Tris-HCl at a pH of 9. Eluted antibodies were buffer exchanged into PBS and concentrated using 50 kDa Amicon Ultra centrifugal filter units.

### Antigen expression and purification

HIV-1 Env antigens were designed with the SOSIP mutations with some modifications. In addition to the SOS (A501C and T605C) and IP (I559P) mutations that form the base of the SOSIP platform, we truncated Envs at position 664. In cases where “DS” is added to the antigen name, the DS (I201C and A433C) mutation set was added. The furin cleavage site between gp120 and gp41 was replaced with a serine-glycine linker (length 15) to create single-chain constructs. The antigens were produced in Expi293F cells by transient transfection in FreeStyle F17 expression media (Thermo Fisher) supplemented to a final concentration of 0.1% Pluronic Acid F-68 and 20% 4mM L-glutamine using Expifectamine transfection reagent (Thermo Fisher Scientific), cultured for 4–7 days at 8% CO_2_ saturation and 37°C with shaking. After transfection, cultures were centrifuged at 4000 g for 20 minutes. Supernatant was filtered with Nalgene Rapid Flow Disposable Filter Units with PES membrane (0.45μm), and then run slowly over a column with agarose-bound Galanthus nivalis lectin (Vector Laboratories cat no. AL-1243-5) at 4°C. The column was washed with PBS, and protein was eluted with 15 mL of 1M methyl-α-D-mannopyranoside. The protein elution was buffer exchanged 3 times into PBS and concentrated using 100kDa Amicon Ultra centrifugal filter units. Concentrated protein was further purified with a Superose 6 Increase 10/300 GL on the AKTA FPLC system. Peaks corresponding to trimeric species were identified based on elution volume.

Influenza HA antigens used in ELISAs for Figs 1 and 2 all contained the HA ectodomain with a point mutation at the sialic acid-binding site (Y98F) and a T4 fibritin foldon trimerization domain. Antigens were expressed in Expi293F mammalian cells using Expifectamine transfection reagent. Cells were cultured for 4–7 days at 8% CO_2_ saturation and 37°C with shaking. After transfection, cultures were centrifuged at 4000 g for 20 minutes. Supernatant was filtered with Nalgene Rapid Flow Disposable Filter Units with PES membrane (0.45μm), and then run slowly over a column with agarose-bound Galanthus nivalis lectin (Vector Laboratories cat no. AL-1243-5) at 4°C. The column was washed with PBS, and protein was eluted with 15 mL of 1M methyl-α-D-mannopyranoside. The protein elution was buffer exchanged 3 times into PBS and concentrated using 50kDa Amicon Ultra centrifugal filter units. Concentrated protein was further purified with Superose 6 Increase 10/300 GL on the AKTA FPLC system. Peaks corresponding to trimeric species were identified based on elution volume.

Influenza HA antigens used in Figs 5 and S1 were produced and purified as previously described [43].

Coronavirus trimeric antigens all had an 8x HisTag along with a TwinStrepTag. SARS-CoV-2 S Hexapro (SARS-CoV-2 HP) encompassed residues 1–1208 of the SARS-CoV-2 spike with a mutated S1/S2 cleavage site, proline substitutions at positions 817, 892, 899, 942, 986, and 987, and a C-terminal T4-fibritin trimerization motif. SARS-CoV spike (SARS-1) encompassed residues 1–1190 of the SARS-CoV spike with proline substitutions at positions 968 and 969, and a C-terminal T4-fibritin trimerization motif. MERS-CoV spike encompassed residues 1–1291 of the MERS-CoV spike with a mutated S1/S2 cleavage site, proline substitutions at positions 1060 and 1061, and a C-terminal T4-fibritin trimerization motif, an AviTag, an 8x HisTag, and a TwinStrepTag. HCoV-HKU1 spike encompassed residues 1–1277 of the HCoV-HKU1 spike with a mutated S1/S2 cleavage site, proline substitutions at positions 1067 and 1068, and a C-terminal T4-fibritin trimerization motif, an 8x HisTag, and a TwinStrepTag. SARS-CoV-2 Omicron BA.2 spike encompassed 1–1208 of the SARS-CoV-2 spike with a mutated S1/S2 cleavage site, proline substitutions at positions 817, 892, 899, 942, 986 and 987, as well as mutations T19I, Del24-26, G142D, V213G, G339D, S371F, S373P, S375F, T376A, D405N, R408S, K417N, N440K, S477N, T478K, E484A, Q493R, Q498R, N501Y, Y505H, D614G, H655Y, N679K, P681H, N764K, D796Y, Q954H, N969K, and a C-terminal T4-fibritin trimerization motif, Avitag, HRV3C, 8x HisTag, and a TwinStrepTag. CoV spikes were purified using StrepTrap XT columns (Cytiva) using the manufacturers instructions.

SARS-CoV-2 NTD glycan knockouts were produced in Expi293 cells and harvested from supernatant on the 6th day post transfection. The NTD containing an 8x-Histidine tag were purified via cobalt affinity chromatography using Talon Metal Affinity Resin (Takara Bio, CA). Supernatant was loaded onto the resin and the resin washed extensively with buffer A (1x HEPES-NaCl pH 7.0) until an absorbance at 280 nm of 0 was reached. The proteins were eluted from the resin by applying a buffer B (1x HEPES-NaCl pH 7.0, 50 mM Imidazole) to the resin. Fractions containing NTD were pooled, concentrated, and further purified by size exclusion chromatography (SEC) using a Superdex 200 Increase 10/300 GL column (Cytiva, MA) equilibrated with 1x HEPES-NaCl pH 7.0. All steps of the purification were performed at room temperature. Protein quality was assessed by SDS-PAGE using NuPage 4–12% (Invitrogen, CA). The purified proteins were flash frozen and stored at −80°C in single-use aliquots. Each aliquot was thawed at 4°C before use.

SARS-CoV-2 spike S1 subunit was obtained from SinoBiological (Cat: 40591-V08H) and was expressed in HEK293 cells. The S1 subunit encompasses amino acids Val16-Arg685.

SARS-CoV-2 spike S2 subunit was obtained from SinoBiological (Cat:40590-V08H1) and was expressed in HEK293 cells. The S2 subunit consisted of the extracellular domain, amino acids 708S-1207E.

SARS-CoV-2 spike RBD subunit was obtained from SinoBiological (Cat:40592-VNAH) and was expressed in HEK293 cells. The RBD subunit consisted of amino acids Arg319-Phe541.

SARS-CoV-2 spike NTD subunit was obtained from SinoBiological (Cat:40591-V49H) and was expressed in HEK293 cells. The NTD subunit consisted of amino acids Met1-Ser305.

HCV E1E2 and E2c containing an AviTag sequence were expressed in Expi293F mammalian cells using Expifectamine transfection reagent and cultured for 4–7 days at 8% CO_2_ saturation and 37°C with shaking. After transfection, cultures were centrifuged at 4000 g for 20 minutes. Supernatant was filtered with Nalgene Rapid Flow Disposable Filter Units with PES membrane (0.45μm), and then run slowly over a column with agarose-bound Galanthus nivalis lectin at 4°C. The column was washed with PBS, and protein was eluted with 15 mL of 1 M methyl-α-D-mannopyranoside. The protein elution was buffer exchanged 3 times into PBS and concentrated. Antigen was further purified with a Superdex200 Increase column.

Parainfluenza antigen containing an 8xHisTag was expressed in Expi 293F mammalian cells using Expifectamine transfection reagent and cultured for 4–7 days at 8% CO_2_ saturation and 37°C with shaking. After transfection, cultures were centrifuged at 4000 g for 20 minutes. Supernatant was filtered with Nalgene Rapid Flow Disposable Filter Units with PES membrane (0.45 μm), and then purified by nickel affinity chromatography using a 1 mL HisTrap HP column. The column was rinsed with binding buffer and purified protein was eluted using binding buffer supplemented with 0.5 M imidazole. The protein elution was buffer exchanged 3 times into PBS and concentrated. Antigen was further purified with a Superose 6 Increase 10/300 GL column.

hMPV F A1 (NL/1/00) and B2 (TN99-419), and RSV F (DS-Cav1) A2 and B9320 antigens were expressed in Expi 293F mammalian cells by transient co-transfection with a 4:1 ratio of F protein to furin using polyethylenimine (PEI). After 6 days the supernatant was filtered and concentrated using tangential flow filtration. Samples were ran over a gravity-flow affinity column at room temperature.

Dengue glycoproteins were recombinantly expressed and purified as previously described [44].

Nipah virus and Langya virus were recombinantly expressed and purified as previously described [26].

### ELISA

ELISAs with HIV-1 Envs, influenza HAs, HCV E, RSV-F, HMPV-F, HPIV-3-F, and Dengue E protein were directly coated onto ELISA plates at a concentration of 2 μg/mL in PBS for overnight at 4°C. Plates were washed three times with PBS-T and then blocked with 1% BSA in PBS-T for one hour at room temperature. Plates were washed three times with PBS-T. A dilution series of antibodies was made in 1% BSA and PBS-T. Plates were washed three times with PBS-T three times, and then antibodies were added. Plates were incubated for one hour at room temperature, and then washed three times with PBS-T. Goat anti-human IgG conjugated to peroxidase was then added at a final dilution of 1:10,000 in 1% BSA and PBS-T. The plates were incubated for one hour at room temperature. Plates were washed three times. Plates were developed using TMB substrate for ten minutes, and the reaction was stopped with 1N sulfuric acid. ELISAs were repeated two additional times.

ELISAs with coronavirus spikes utilized a streptavidin capture strategy. Streptavidin was plated overnight at 2 μg/mL at 4°C. Excess streptavidin was washed away three times with PBS supplemented with 0.05% Tween20 (PBS-T) and blocked with 1% BSA in PBS-T. Plates were incubated for one hour and then washed three times with PBS-T. Coronavirus spikes were diluted to 2 μg/mL in 1% BSA and PBS-T and then added to the plates. Plates were incubated for two hours at room temperature and then washed three times with PBS-T. All other antigens utilized a direct coating strategy, whereby antigens were plated overnight at 2 μg/mL at 4°C, followed by blocking as described above. A dilution series of antibodies was made in 1% BSA in PBS-T and then added to plates. Plates were incubated for one hour and then washed three times with PBS-T. Goat anti-human IgG conjugated to peroxidase was then added at a final dilution of 1:10,000 in 1% BSA and PBS-T. The plates were incubated for one hour at room temperature. Plates were washed three times. Plates were developed using TMB substrate for ten minutes, and the reaction was stopped with 1N sulfuric acid. ELISAs were repeated two additional times.

### Glycan-dependent binding of antibodies to antigens

Glycan-dependency of antibodies was assessed by treating antigens under different conditions followed by ELISA. Antigens under the “native” condition had no treatment. Antigens under the "37°C” condition were diluted in 1X Glycobuffer 2 and incubated at 37°C overnight. Antigens under the "37°C+PNGase-F” condition were diluted in 1X Glycobuffer 2 and incubated with PNGase-F (New England Biolabs) at 37°C overnight. Antigens under the “denature” condition were diluted in Glycoprotein Denaturing Buffer (New England Biolabs) and boiled at 90°C for 10 minutes. Antigens in the “1 M mannose competition” condition had no treatment. Antigens were then directly coated onto ELISA plates at a concentration of 2 μg/mL in PBS for two hours at room temperature. Plates were washed three times with PBS-T and then blocked with 1% BSA in PBS-T for one hour at room temperature. Plates were washed three times with PBS-T. A dilution series of antibodies was made in 1% BSA and PBS-T. Antibodies in the “1M Mannose Competition” condition has 1 M mannose present in the dilution buffer and were allowed to sit for one hour prior to addition to the plate. Plates were washed three times with PBS-T three times, and then antibodies were added. Plates were incubated for one hour at room temperature, and then washed three times with PBS-T. Goat anti-human IgG conjugated to peroxidase was then added at a final dilution of 1:10,000 in 1% BSA and PBS-T. The plates were incubated for one hour at room temperature. Plates were washed three times. Plates were developed using TMB substrate for ten minutes, and the reaction was stopped with 1N sulfuric acid. ELISAs were repeated two additional times.

### Competition ELISA

Competition ELISAs were performed as described above for HIV-1 Env antigens with minor modifications. After coating with antigen and blocking, non-biotinylated competitor antibody was added to each well at 10 μg/mL and incubated at RT for one hour. Without washing, biotinylated antibody (final concentration 10 μg/mL) was added and incubated at RT for one hour. After washing three times with PBS-T, streptavidin-HRP was added at 1:10,000 dilution in 1% BSA in PBS-T and incubated for one hour at RT. Plates were then washed and substrate and sulfuric acid were added as described above.

### Anti-nuclear Ab (ANA) reactivity

Antibody reactivity to nine human autoantigens was measured using the AtheNA Multi-Lyte ANA kit (Zeus scientific, Inc, #A21101). Antibodies were serially diluted for four 2-fold steps starting at 300 μg/mL. The antibodies were then diluted 1:6 in assay beads resulting in a final serial dilution of four 2-fold steps starting at 50 mcg/mL in 50ul of beads. The kit SOP was followed for the remainder of the assay. Samples were analyzed using AtheNA software. An individual well was positive if >120 AU (Athena Units), negative if <100 and considered indeterminate if between 100–120. An antibody needed to be positive for two consecutive wells (i.e. to at least 25 μg/ml) to be considered positive for autoreactivity.

### HEp-2 cell staining

HEp-2 cell coated slides (BION ENTERPRISES LTD ANA (HEp-2) Test System, ANK-120) were incubated with purified antibodies at 100, 10, or 1 μg/ml or control sera in a moist chamber at room temperature for 30 min. Controls provided with the kit included anti-nuclear antibody (ANA)+ and ANA- human sera. Slides were washed twice with PBS for 5 min. Cells were stained with FITC-goat anti-human Ig per the manufacturer’s instructions and incubated in a moist chamber at room temperature for 30 min. Slides were washed twice with PBS for 5 min, mounted with DAPI mounting medium (Southern Biotech 0100–20) and visualized by fluorescence microscopy (Olympus BX60 epifluorescence microscope coupled with a CCD camera and MagnaFire software (Optronics International) at 40x magnification. Image brightness and contrast were optimized using Adobe Photoshop.

### Surface Plasmon Resonance (SPR)

Binding experiments were performed using SPR on a Biacore T-200 (Cytiva, MA) with HBS buffer supplemented with 3 mM EDTA and 0.05% surfactant P-20 (HBS-EP+, Cytiva, MA). All binding assays were performed at 25°C. Antigen binding to antibodies was assessed using a Series S CM5 chip (Cytiva, MA) which was labeled with anti-human IgG (fc) antibody using a Human Antibody Capture Kit (Cytiva, MA). IgG’s were coated at 200 nM (120s at 5 μL/min) on the chip surface. For single injection experiments, NiV-F or LayV-F was injected at 50 nM over the antibodies using the high-performance injection mode (240 sec on, 540 sec off, 50 μL/min). BG505.DS.SOSIP or BG505.SOSIP was injected at 50 nM over the antibodies using the high-performance injection mode (240 sec on, 540 sec off, 50 μL/min). SARS-CoV was injected at 100 nM over the antibodies using the high-performance injection mode (240 sec on, 540 sec off, 50 μL/min). The surface was regenerated three pulses of a 3 M MgCl2 solution for 10 seconds at 100μL/min. Brisbane/2007 HA (H3), Michigan/2016 HA (H1), Taiwan/2013 HA (H7), or Turkey/2005 HA (H5) was injected at 100 nM over the antibodies using the high-performance injection mode (240 sec on, 540 sec off, 50 μL/min). SARS-CoV was injected at 100 nM over the antibodies using the high-performance injection mode (240 sec on, 540 sec off, 50 μL/min). Sensogram data were analyzed using the BiaEvaluation software (Cytiva, MA)

### 2526 IgG1 Fab purification

Purified 2526 IgG1 antibody were subsequently digested to its Fab state using LysC. The Fab was further purified using a Protein A spin column to remove undigested antibody and the Fc portion. Fab was applied to a Superdex 200 10/300 GL Increase column and fractions containing the Fab were pooled and concentrated using an Amicon Ultra-15 Centrifugal filter 3 kDa MWCO (Millipore). Protein purity was assessed by SDS-PAGE using NuPage 4–12% (Invitrogen, CA).

### Negative stain electron microscopy

A frozen aliquot from -80°C was thawed at RT in an Al block for 5 min. Sample was then diluted to 20 μg/ml with 0.02 g/dl Ruthenium Red in HBS (20 mM HEPES, 150 mM NaCl pH 7.4) buffer. After 10–15 min incubation, sample was applied to a glow-discharged carbon-coated EM grid for 8–10 second, blotted, consecutively rinsed with 2 drops of 1/20X HBS, and stained with 2 g/dL uranyl formate for 1 min, blotted and air-dried. Grids were examined on a Philips EM420 electron microscope operating at 120 kV and nominal magnification of 49,000x, and **25** images were collected on a 76 Mpix CCD camera at 2.4 Å/pixel. Images were analyzed by 2D class averages using standard protocols with Relion 3.0 [45].

### Virus neutralization assays

Neutralization of HIV-1 pseudoviruses was assessed using the TZM-bl neutralization assay as described previously [46]. Viruses that represent circulating strains (tier 2) were included. Neutralization was measured as a reduction in luciferase gene expression after a single round of infection of TZM-bl cells in a 96-well plate. Results are presented as antibody concentration required to inhibit 50% of virus infection (IC_50_).

SARS-CoV-2 lentivirus-based pseudoviral neutralization assays were executed based on a previous described protocol [47]. One day prior to the assay, HEK-293T cells transduced to stably express human ACE2 (293T-hACE2 cells, BEI Resources NR-52511) were seeded at a density of 1.25x10^4^ cells per well in DMEM+10% FBS onto 96-well tissue culture plates that had previously been coated with poly-D-lysine (ThermoFisher Scientific, cat. A3890401) The day of the assay, monoclonal antibodies were diluted in DMEM+2% FBS using a 4-fold dilution series (with each antibody in technical duplicate) in a 96-well polypropylene microtiter plate and incubated with pseudovirus for 1 h at 37°C in the presence of a final concentration of 5 mg/mL polybrene (EMD Millipore). After the 1 h incubation, pseudovirus-mAb mixtures were added to 293T-hACE2 monolayers. Plates were incubated at 37°C for 48–60 h, at which point cells were lysed using the Bright-Glo Luciferase Assay System (Promega) and luciferase activity was quantified using a CLARIOStar plate reader (BMG LabTech). The luminescence signal from wells in each plate where no pseudovirus or antibody was added was averaged and subtracted from each value, after which the percent infection of each well was determined relative to the average of pseudovirus only control wells present in each plate. IC50 values were determined by nonlinear regression using Prism v.9.5 (GraphPad) using a four-parameter [inhibitor] vs. response curve fit with top and bottom values constrained to 100 and 0, respectively. Each neutralization assay was repeated twice.

Influenza virus neutralization assays were performed as previously described [48].

### Animal studies

BALB/cJ mice (female, 6–8 week of age), antibody negative for circulating influenza A (H1N1 and H3N2) and influenza B viruses, were purchased from Jackson Laboratories (Bar Harbor, ME) and housed in microisolator units and fed ad libitum. Mice were handled in accordance with protocols approved by the Cleveland Clinic Institutional Animal Care and Use Committee and were cared under U.S. Department of Agriculture guidelines for laboratory animals. Animals were acclimated to study housing for at least 72 hours prior to initiation of the studies. Mice that showed signs of severe morbidity or lost >25% of their original weight were humanly euthanized.

Mice were grouped into 7 groups of 8 mice each. Each experimental group was administered with either 2526 IgG3 mAb or an isotype control mAb (71282–18) at a 20 mg/kg dose per mouse. Mab 71282–18 is an IgG3 antibody specific for CoV spikes [10]. Mab administrations were preformed intraperitoneally 24 hours before or 24 hours after treatment IAV infection for the prophylactic and therapeutic groups, respectively. A control group immunized with PBS was run in tandem with the experimental groups. All mice were inoculated intranasally with H1N1 A/Brisbane/01/2018 influenza virus strain at a dose of 3.6x10^6^ PFU and weight loss was tracked for 14 days post infection challenge. Lungs were collected 3 days post challenge from 3 mice per group to assess the lung viral titer.

Lung viral titer: for lung virus tittering, a plaque assay was performed similarly to previously described protocols (REF). In brief, low-passage MDCK cells were plated at a confluency of 3.8 × 105 in each well of a six-well plate (Greiner Bio-One, Monroe, NC) 2 days before the assay. Samples were diluted and overlaid onto the cells in 100 μL of DMEM supplemented with penicillin-streptomycin and incubated for 1 h. Samples were removed, cells were washed, and medium was replaced with 2 mL of Modified Eagle Medium plus 0.8% agarose (Cambrex, East Rutherford, NJ) and incubated for 72 h at 37°C with 5% CO2. Agarose was removed and discarded. The cells were then fixed with 10% buffered formalin and then stained with 1% Crystal Violet (Fisher Science Education) for 15 min. Following thorough washing in distilled water to remove excess Crystal Violet, the plates were dried, the numbers of plaques counted, and the PFU per milliliter or per gram of lung tissue was calculated.

### Western blots

Western blots for virus-derived and recombinant HA were performed as previously described [48]. PN-SIA49 was used as a positive control antibody [49,50].

### Proteolytic digestion of BG505

BG505.T332N.SOSIP and BG505.T332N.DS.SOSIP samples (80 μg) at a concentration of 2.5 mg/mL were denatured with 7 M urea in 100 mM Tris buffer (pH 8.0), reduced at RT for one hour with TCEP (5 mM), and alkylated with 20 mM IAM at RT for another hour in the dark. The reduced and alkylated samples were buffer exchanged with 50 mM ammonium bicarbonate (pH 8) using a 30-kDa MWCO filter (Millipore) prior to trypsin digestion. Digestion was performed using trypsin at a 30:1 protein:enzyme ratio. Samples were incubated overnight at 37°C. A 20 μL aliquot of the tryptic digest was treated with chymotrypsin and was incubated at 37°C for in the dark for eight hours. The resulting trypsin and trypsin/chymotrypsin digests were either directly analyzed or stored at -20°C until further analysis.

### Chromatography and mass spectrometry

High-resolution LC/MS experiments were performed using an Orbitrap Fusion Lumos Tribrid (Thermo Scientific) mass spectrometer equipped with ETD that is coupled to an Acquity UPLC M-Class system (Waters). Mobile phases consisted of solvent A: 99.9% deionized H_2_O + 0.1% formic acid and solvent B: 99.9% CH_3_CN + 0.1% formic acid. Three microliters of the sample were injected onto C18 PepMap 300 column (300 μm i.d. x 15 cm, 300 Å, Thermo Fisher Scientific) at a flow rate of 3 μL/min. The following CH_3_CN/H_2_O multistep gradient was used: 3% B for 5 min, followed a linear increase to 40% B in 50 min, then a linear increase to 90% B in 15 min. The column was held at 97% B for 10 minutes before re-equilibration. All mass spectrometric analysis was performed in the positive ion mode using data-dependent acquisition with the instrument set to run in 3-sec cycles for the survey and two consecutive MS/MS scans with CID and ETD. The full MS survey scans were acquired in the Orbitrap in the mass range, 350–1900 *m/z* at a resolution of 120000 at *m/z* 200 with an AGC target of 4 x10^5^ and a maximum injection time of 50 ms. Following a survey scan, MS/MS scans were performed on the most intense ions with charge states ranging from 2–6 and with intensity greater than 5000. CID was carried out at with a collision energy of 30% while ETD was performed using the calibrated charge dependent reaction time. Resulting fragments were detected using rapid scan rate in the ion trap.

### Glycopeptide identification

Details of the glycopeptide compositional analysis have been described previously [51–53]. Briefly, compositional analysis of glycopeptides was carried out by first identifying the peptide portion from tandem MS data. Once the peptide portion was determined, plausible glycopeptide compositions were obtained using the high-resolution MS data and GlycoPep DB. The putative glycopeptide composition was confirmed manually from CID and ETD data.

### Mass photometry

Mass determination of the glycoproteins was conducted using a Refeyn TwoMP mass photometer (Oxford, UK). PBS buffer was used for instrument focusing. Mass calibrations were performed using ß-amylase (Sigma-A8781), Apoferritin (Sigma-A3660), and Thyroglobulin (Sigma-T9145) at final concentrations of 5 nM. Mass calibration yielded a standard mass curve with an R-squared of 1.0 and a mass error of 7.1%. Glycoproteins at 20 nM were diluted to a final concentration of 2 nM for mass determination readings. Data were recorded using the AcquireMP software and analyzed and fitted using the DiscoverMP software (Refeyn, Oxford, UK).

### X-ray crystallography

Crystals were grown via the vapor diffusion method at room temperature in siting drop well format. 2526 Fab crystals were formed by mixing 0.25 uL of 2526 Fab at 40 mg/mL and 1 M D-Mannose with 0.15 uL of well solution containing 75 mM HEPES pH 7.5, 10% PEG10K, and 25% glycerol. Crystals appeared within one week. Cryoprotection of the crystals was done by mixing glycerol to a final concentration of 50% with the well containing the crystals along with 1 M D-Mannose. Crystals were flash-cooled in liquid nitrogen and data were collected at the NSLS-II, Beamline 19-ID at 100 K and a wavelength of 1 Å. All data were indexed and integrated using iMosflm and scaled using AIMLESS [54,55]. Phaser in the PHENIX suite was used to per- form molecular replacement using PDB 72CL as a search model [56]. Phenix.refine was used for data refinement, and manual refinement was done in Coot [57–59].

## Supporting information

S1 Fig Expanded breadth panel of 2526 against H5, H2, and H6 HAs. 2526 binding was measured against several additional HAs in an ELISA format. Strains with avian names indicate isolation from an animal reservoir whereas strains with a location name indicate isolation from a human reservoir.
(TIF)

S2 Fig SPR data for glycan-reactive antibodies. (A) 2526, 546, and 688 were measured for binding to NiV-F and LayV-F. (B) 2526, 546, and 688 were tested for differential binding between antigens made in 293F cells or GnT1- cells. Representative curves from a set of three repeats are shown.
(TIF)

S3 Fig Mass photometry of antigens produced in 293F or GnT1- cells. Mass photometry of glycoproteins made in either 293F cells or GnT1- cells was conducted to assess the size of each antigen. Antigens made in GnT1- cells lack processed N-linked glycans, thus giving a smaller size.
(TIF)

S4 Fig Glycosylation profiles of BG505 Env glycoproteins. Bar graphs showing the glycan profiles at each identified glycosylation site. The glycan compositions (in percent) were broadly categorized into two classes: high-mannose (red bar) and processed glycans (blue bar).
(TIF)

S5 Fig Stabilization of 2526 IgG1 light chain glycan binding pocket. The observed glycan binding pocket of 2526 IgG1 Fab is maintained by hydrogen bonding interactions between the CDR2 loop of the light chain and residues of the pocket. Specifically, the backbone carbonyls of V58 and P59 are interacting with the epsilon nitrogen of the R54. R54 is also forming another hydrogen bond with the backbone carbonyl of P60 and the NH_2_ of the side chain.
(TIF)

S6 Fig 2526 and 546 perform better when expressed in their native IgG3 isotype. 2526 and 546 were expressed as either IgG1 or IgG3 and tested for binding against influenza HA (New Caledonia/1999 H1), HIV-1 Env (KNH1209.18.DS.SOSIP), and SARS-CoV-2 spike (index strain) in an ELISA format. In all cases, the IgG3 versions outperformed the IgG1 versions.
(TIF)

S7 Fig Additional epitope mapping of 2526 for HIV-1 Env. (A) 2526 binding to various HIV-1 Env constructs was tested in ELISA under different conditions of antigen treatment. (B) 2526 bound equally well to all mutants of an HIV-1 Env trimer (KNH1209.18.DS.SOSIP) where individual glycans were knocked out, as well as to a trimer where all glycans in the V1V2 region were simultaneously knocked out (V1V2 Glycan KO).
(TIF)

S8 Fig Western blot of 2526 binding to HAs purified from virus or recombinantly produced. (A) 2526 (left) and mAb PN-SIA49 (right) binding to recombinantly produced HAs under non-reducing or reducing (R) conditions. (B) 2526 (left) and mAb PN-SIA49 (right) binding to HAs purified from infectious virions following split inactivation of the viruses under non-reducing or reducing (R) conditions. (C) SDS-PAGE of the corresponding recombinant HAs and viruses used in the western blots. Bands corresponding to HA fall between 70–100 kDa.
(TIF)
