# Supplementary figures and images for: Isolation and characterization of IgG3 glycan-targeting antibodies with exceptional cross-reactivity for diverse viral families
Source: PLoS Pathog. 2024 Sep 18;20(9):e1012499. doi: 10.1371/journal.ppat.1012499 (PMC11410209; doi:10.1371/journal.ppat.1012499)

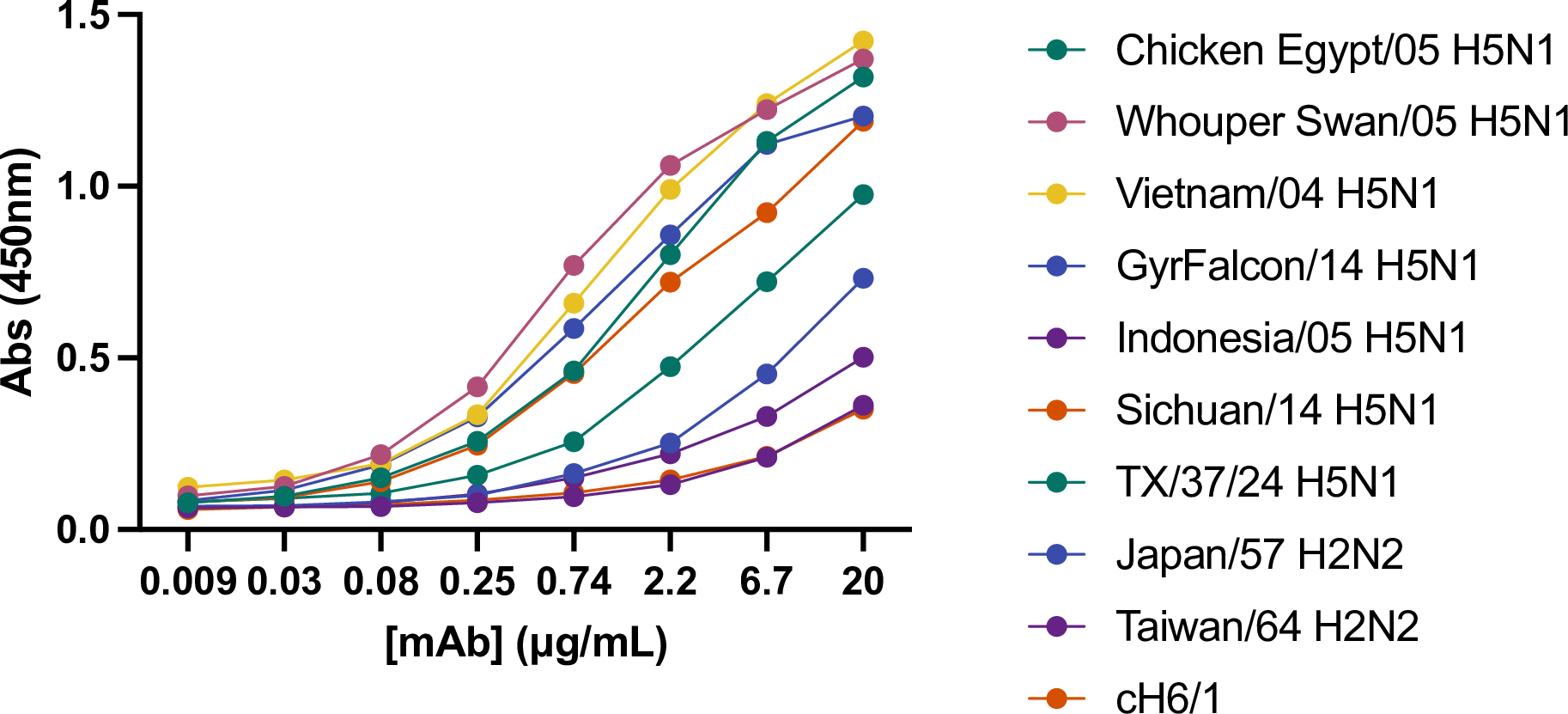

Supplement: S1 Fig — 2526 binding was measured against several additional HAs in an ELISA format. Strains with avian names indicate isolation from an animal reservoir whereas strains with a location name indicate isolation from a human reservoir. (TIF) [file ppat.1012499.s001.tif]

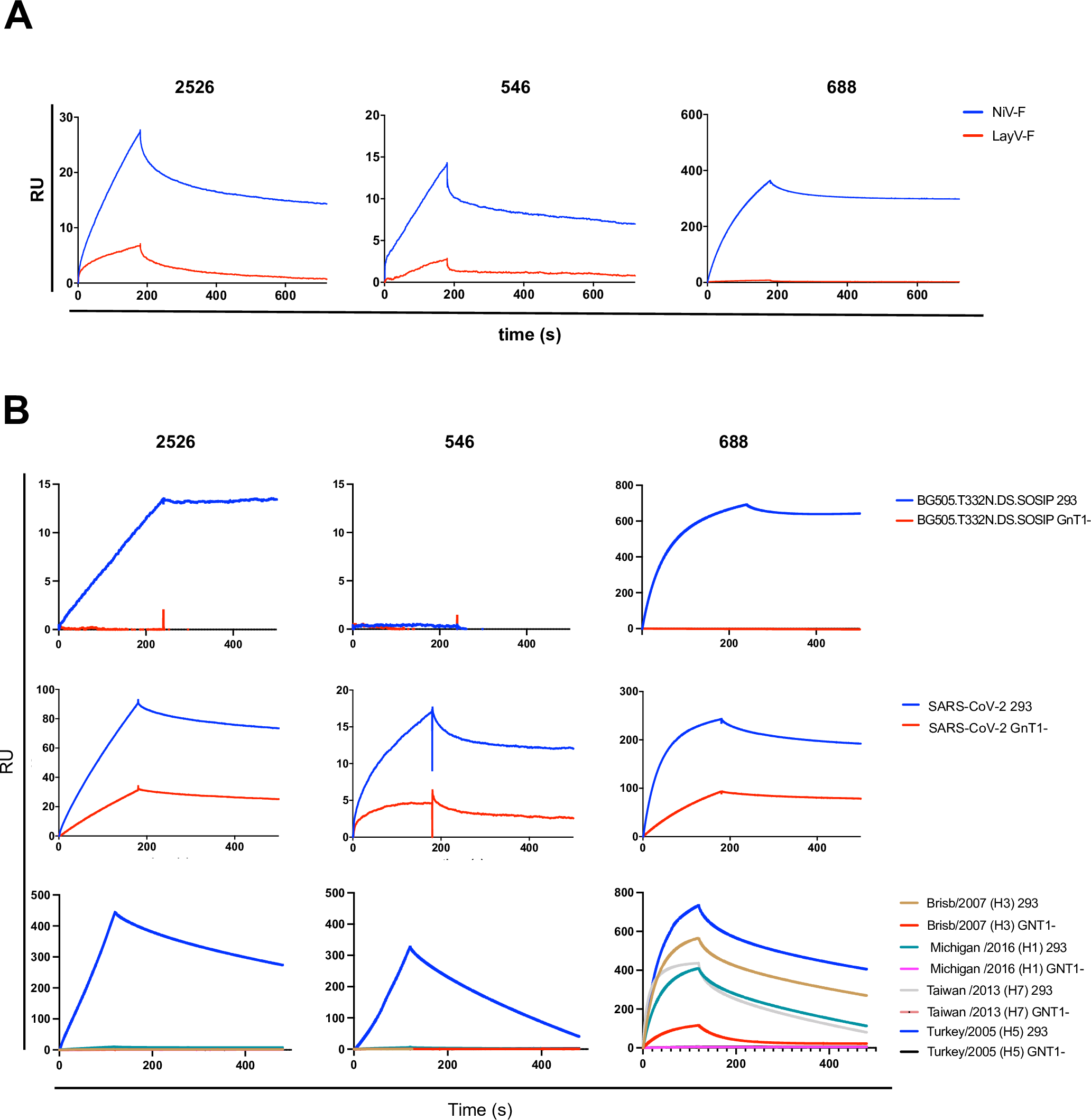

Supplement: S2 Fig — (A) 2526, 546, and 688 were measured for binding to NiV-F and LayV-F. (B) 2526, 546, and 688 were tested for differential binding between antigens made in 293F cells or GnT1- cells. Representative curves from a set of three repeats are shown. (TIF) [file ppat.1012499.s002.tif]

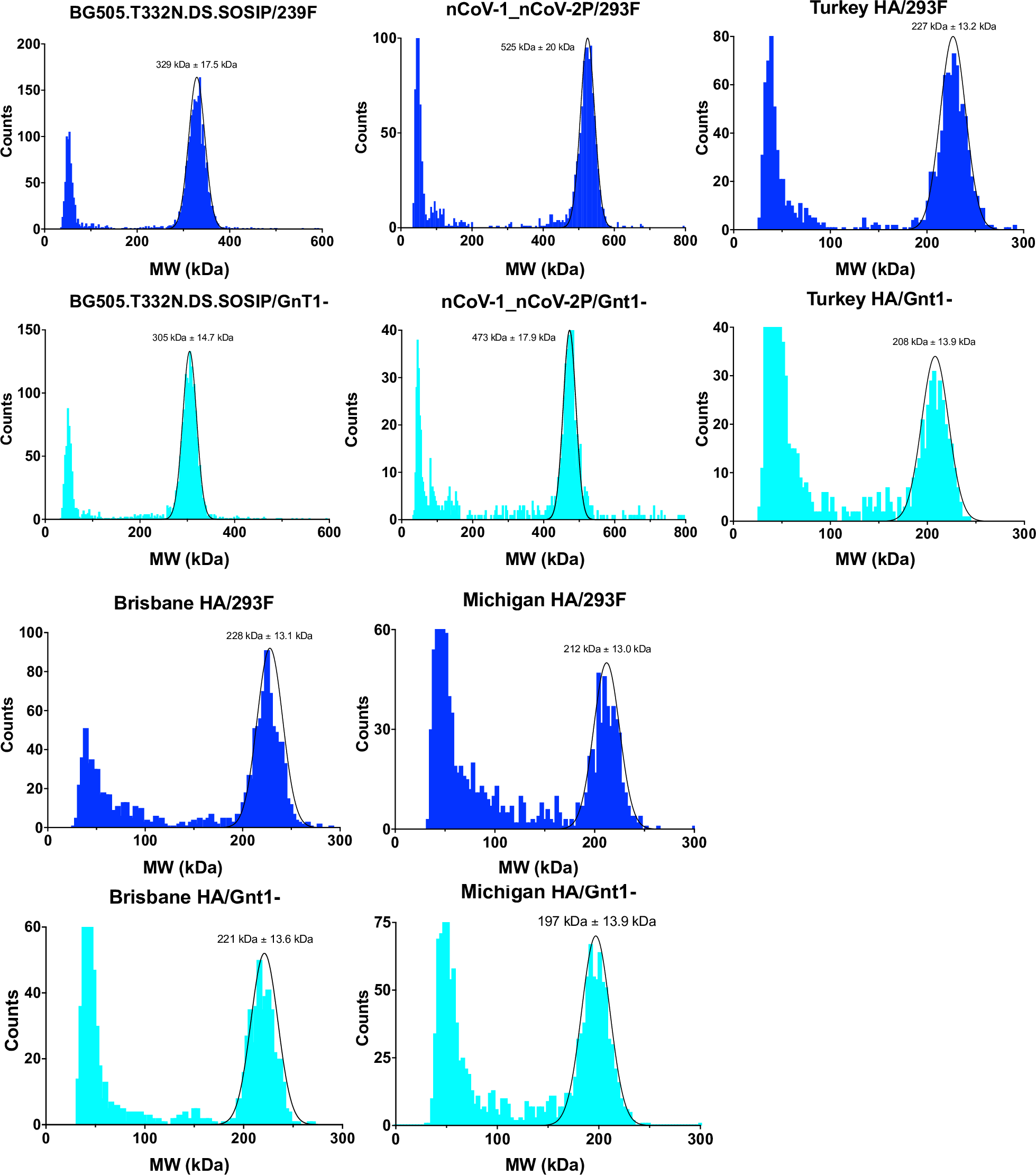

Supplement: S3 Fig — Mass photometry of glycoproteins made in either 293F cells or GnT1- cells was conducted to assess the size of each antigen. Antigens made in GnT1- cells lack processed N-linked glycans, thus giving a smaller size. (TIF) [file ppat.1012499.s003.tif]

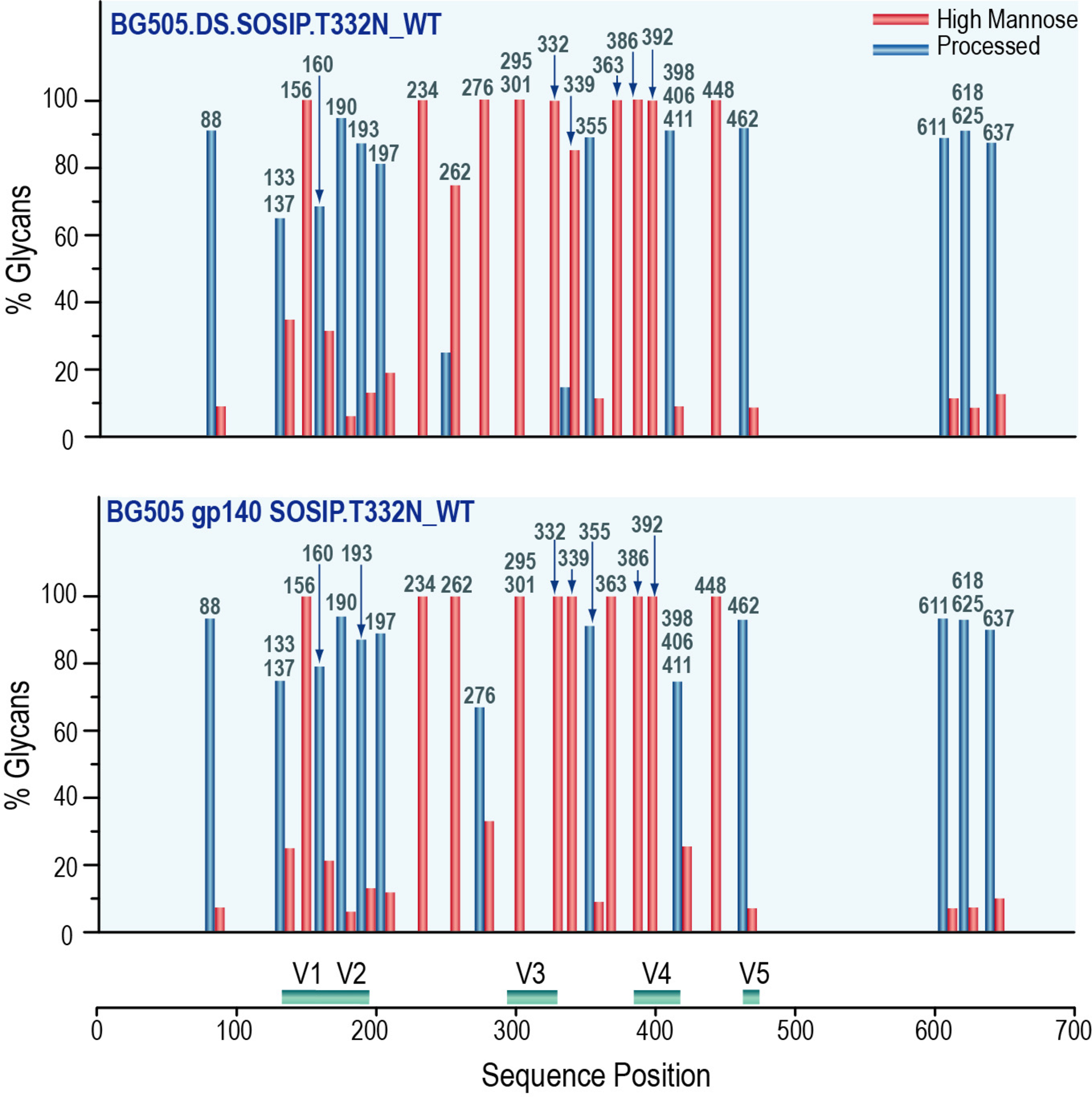

Supplement: S4 Fig — Bar graphs showing the glycan profiles at each identified glycosylation site. The glycan compositions (in percent) were broadly categorized into two classes: high-mannose (red bar) and processed glycans (blue bar). (TIF) [file ppat.1012499.s004.tif]

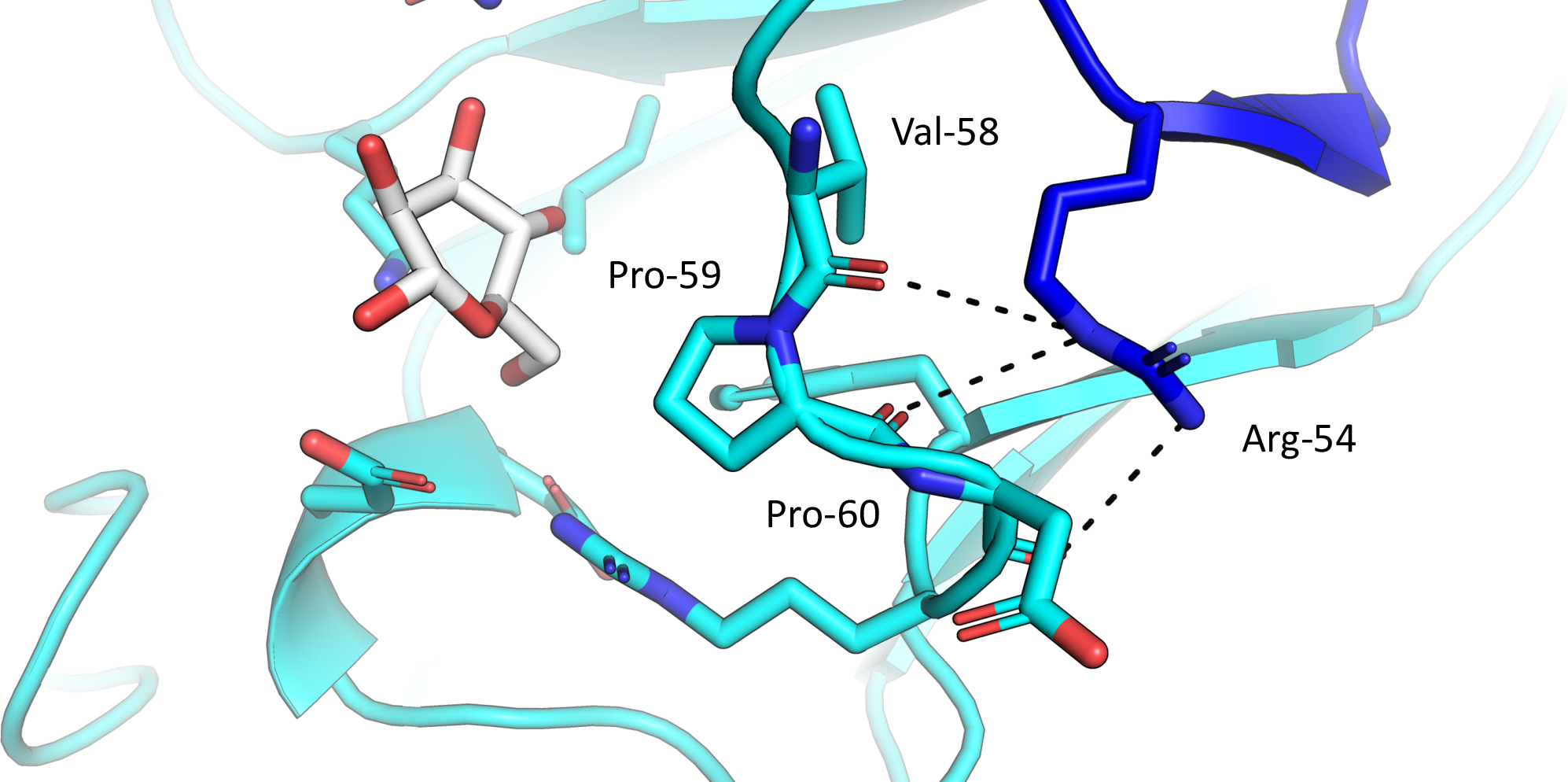

Supplement: S5 Fig — The observed glycan binding pocket of 2526 IgG1 Fab is maintained by hydrogen bonding interactions between the CDR2 loop of the light chain and residues of the pocket. Specifically, the backbone carbonyls of V58 and P59 are interacting with the epsilon nitrogen of the R54. R54 is also forming another hydrogen bond with the backbone carbonyl of P60 and the NH2 of the side chain. (TIF) [file ppat.1012499.s005.tif]

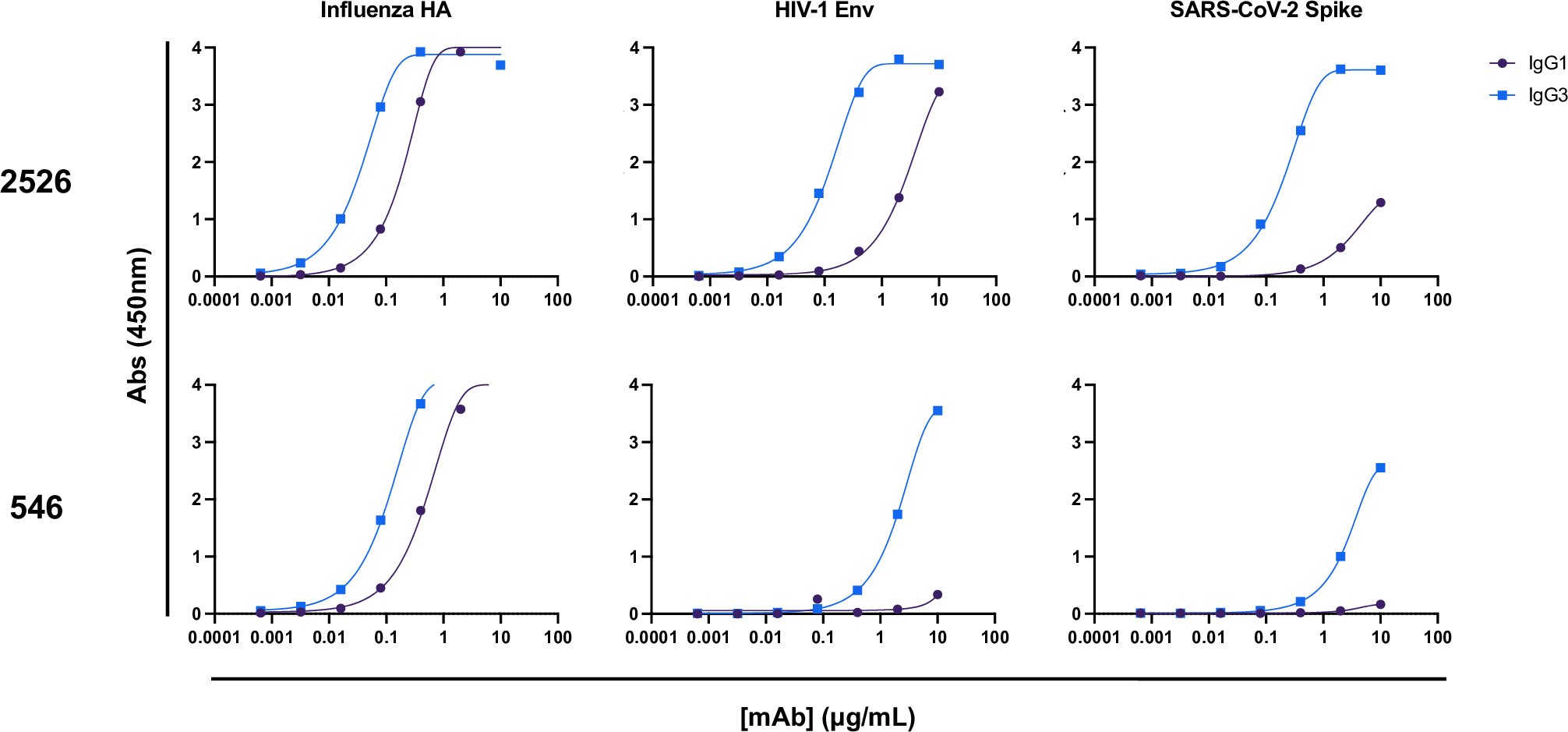

Supplement: S6 Fig — 2526 and 546 were expressed as either IgG1 or IgG3 and tested for binding against influenza HA (New Caledonia/1999 H1), HIV-1 Env (KNH1209.18.DS.SOSIP), and SARS-CoV-2 spike (index strain) in an ELISA format. In all cases, the IgG3 versions outperformed the IgG1 versions. (TIF) [file ppat.1012499.s006.tif]

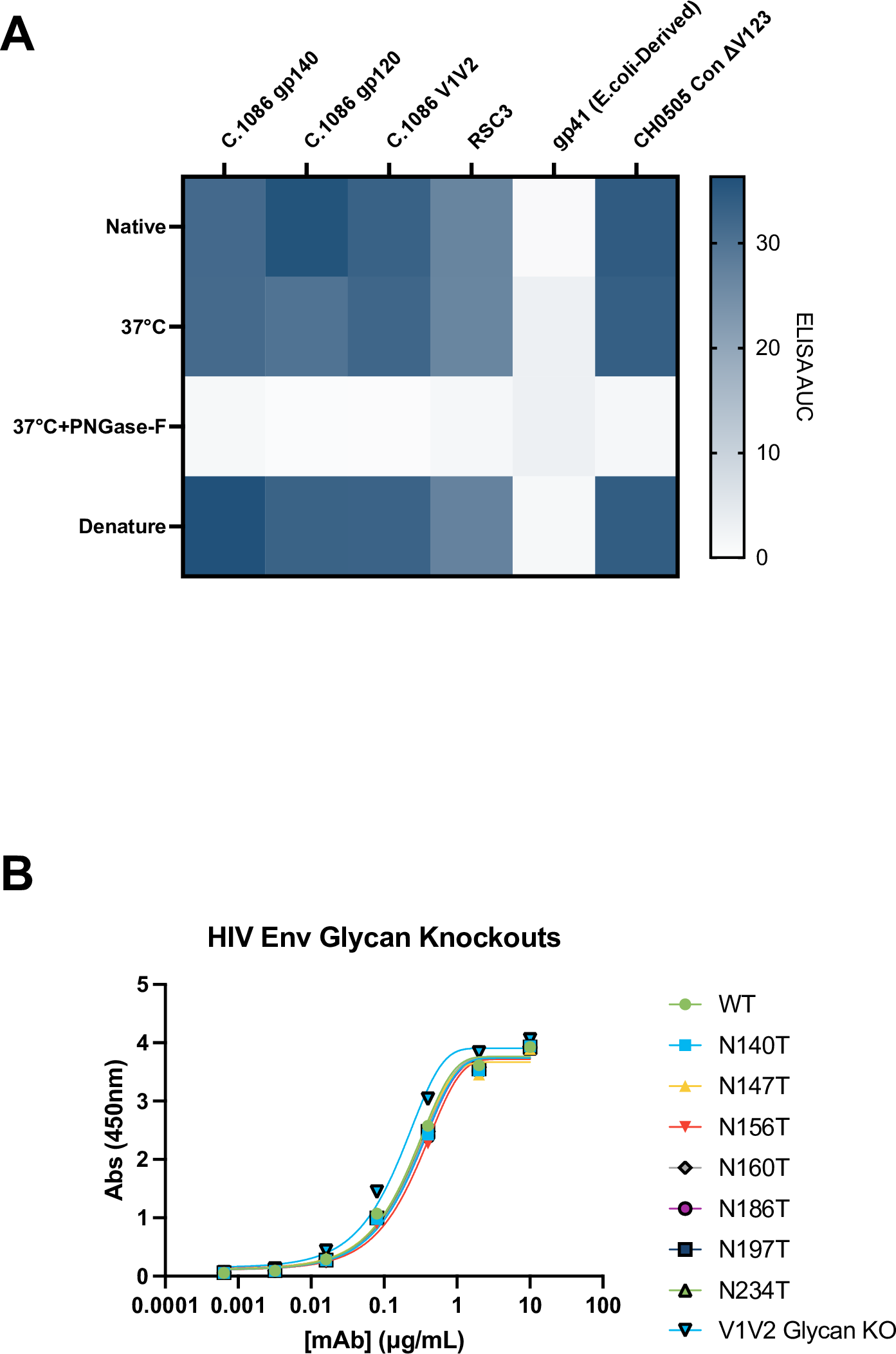

Supplement: S7 Fig — (A) 2526 binding to various HIV-1 Env constructs was tested in ELISA under different conditions of antigen treatment. (B) 2526 bound equally well to all mutants of an HIV-1 Env trimer (KNH1209.18.DS.SOSIP) where individual glycans were knocked out, as well as to a trimer where all glycans in the V1V2 region were simultaneously knocked out (V1V2 Glycan KO). (TIF) [file ppat.1012499.s007.tif]

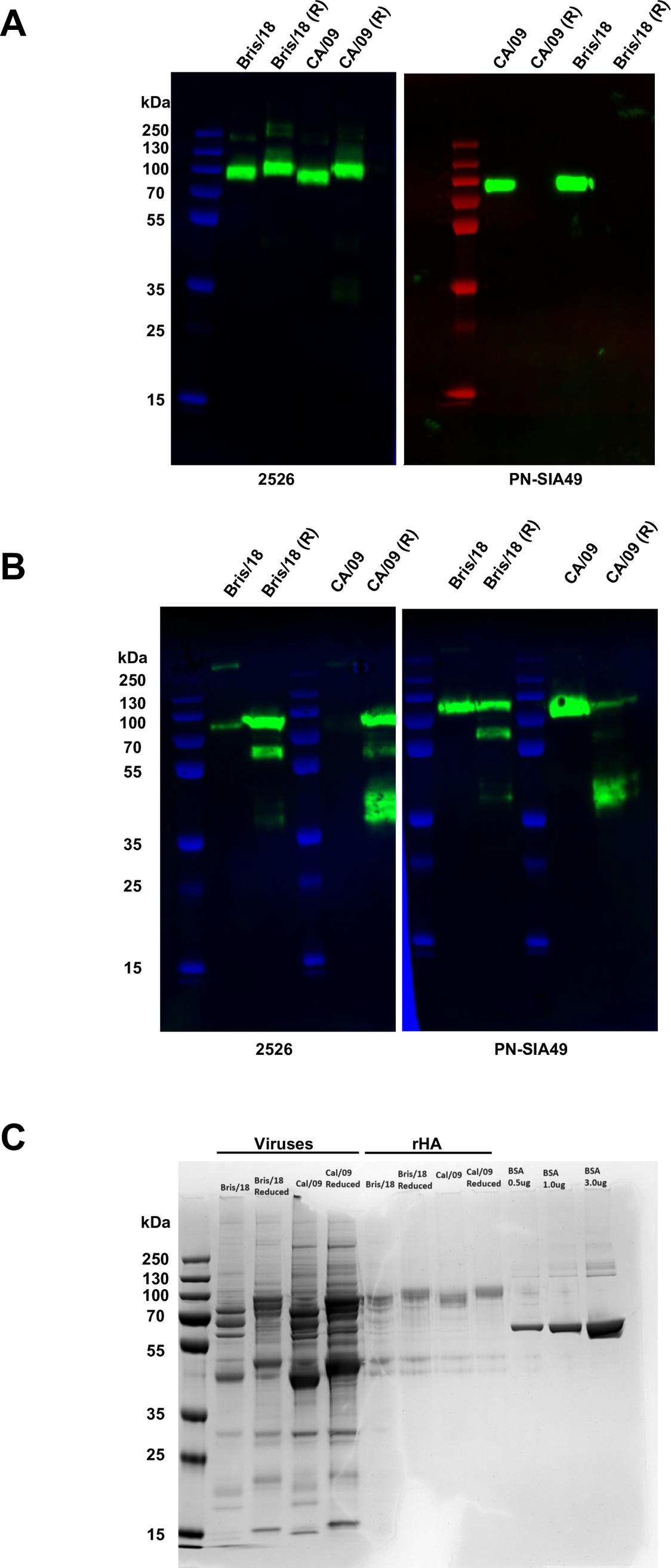

Supplement: S8 Fig — (A) 2526 (left) and mAb PN-SIA49 (right) binding to recombinantly produced HAs under non-reducing or reducing (R) conditions. (B) 2526 (left) and mAb PN-SIA49 (right) binding to HAs purified from infectious virions following split inactivation of the viruses under non-reducing or reducing (R) conditions. (C) SDS-PAGE of the corresponding recombinant HAs and viruses used in the western blots. Bands corresponding to HA fall between 70–100 kDa. (TIF) [file ppat.1012499.s008.tif]
